# Large-Scale Screening of 239 Traditional Chinese Medicinal Plant Extracts for Their Antibacterial Activities against Multidrug-Resistant *Staphylococcus aureus* and Cytotoxic Activities

**DOI:** 10.3390/pathogens9030185

**Published:** 2020-03-04

**Authors:** Gowoon Kim, Ren-You Gan, Dan Zhang, Arakkaveettil Kabeer Farha, Olivier Habimana, Vuyo Mavumengwana, Hua-Bin Li, Xiao-Hong Wang, Harold Corke

**Affiliations:** 1 Department of Food Science & Technology, School of Agriculture and Biology, Shanghai Jiao Tong University, Shanghai 200240, China; gowoon_kim@sjtu.edu.cn (G.K.); zhang.dan@sjtu.edu.cn (D.Z.); farhatintu@sjtu.edu.cn (A.K.F.); 2 Research Center for Plants and Human Health, Institute of Urban Agriculture, Chinese Academy of Agricultural Sciences, Chengdu 610213, China; 3 School of Biological Sciences, The University of Hong Kong, Hong Kong 999077, China; ohabim@hku.hk; 4 DST/NRF Centre of Excellence for Biomedical Tuberculosis Research, US/SAMRC Centre for Tuberculosis Research, Division of Molecular Biology and Human Genetics, Department of Biomedical Sciences, Faculty of Medicine and Health Sciences, Stellenbosch University, Cape Town 8000, South Africa; vuyom@sun.ac.za; 5 Guangdong Provincial Key Laboratory of Food, Nutrition and Health, Department of Nutrition, School of Public Health, Sun Yat-Sen University, Guangzhou 510080, China; lihuabin@mail.sysu.edu.cn; 6 College of Food Science and Technology, Huazhong Agricultural University, Wuhan 430070, China; wxh@mail.hzau.edu.cn

**Keywords:** medicinal plant, antimicrobial activity, drug resistance, foodborne pathogens, cytotoxicity, polyphenols

## Abstract

Novel alternative antibacterial compounds have been persistently explored from plants as natural sources to overcome antibiotic resistance leading to serious foodborne bacterial illnesses. In this study, the ethanolic extracts from 239 traditional Chinese medicinal plants (TCMP)’ materials were screened to discover promising candidates that have strong antibacterial properties against multidrug-resistant *Staphylococcus (S.) aureus* and low cytotoxicity. The results revealed that 74 extracts exhibited good antibacterial activities (diameter of inhibition zone (DIZ) ≥ 15 mm). Furthermore, 18 extracts (DIZ ≥ 20 mm) were determined their minimum inhibitory concentrations (MIC) and minimum bactericide concentrations (MBC), ranging from 0.1 to 12.5 mg/mL and 0.78 to 25 mg/mL, respectively. In addition, most of the 18 extracts showed relatively low cytotoxicity (a median lethal concentration (LC_50_) >100 µg/mL). The 18 extracts were further determined to estimate possible correlation of their phenolic contents with antibacterial activity, and the results did not show any significant correlation. In conclusion, this study selected out some promising antibacterial TCMP extracts with low cytotoxicity, including *Rhus chinensis* Mill., *Ilex rotunda* Thunb., *Leontice kiangnanensis* P.L.Chiu, *Oroxylum indicum* Vent., *Isatis tinctorial* L., *Terminalia chebula* Retz., *Acacia catechu* (L.f.) Willd., *Spatholobus suberectus* Dunn, *Rabdosia rubescens* (Hemsl.) H.Hara, *Salvia miltiorrhiza* Bunge, *Fraxinus fallax* Lingelsh, *Coptis chinensis* Franch., *Agrimonia Pilosa* Ledeb., and *Phellodendron chinense* C.K.Schneid.

## 1. Introduction

Foodborne diseases are mainly transmitted by bacterial-contaminated food and pose serious public health problems that cause significant morbidity and mortality worldwide [1]. According to the World Health Organization (WHO) report, approximately 2.2 million people annually die from foodborne diseases caused by pathogens [2]. *Staphylococcus* (*S.*) *aureus*, one of the major foodborne pathogens, leads to a wide range of severe foodborne diseases due to its invasiveness, multidrug resistance, and virulence [3,4]. The indiscriminate use of antibiotics in animal husbandry and hospitals have accelerated the emergence of *S. aureus* to acquire resistance to various antibacterial agents [5,6,7,8]. The rapid development of multidrug resistance has led to a decrease in the effectiveness of commercial antibiotics and has resulted in untreatable infections [9]. Moreover, a challenging complication is presented by the slow pace of research and development of new antibiotics that may add to the existing arsenal of antimicrobials to circumvent the outbreak and transmission of drug resistant bacteria [10]. As such, to overcome the outbreak and proliferation of multidrug-resistant bacterial infections, novel antibacterial compounds of plant, animal, bacterial, algal, and fungal origin have been persistently explored as an alternative to conventional antibiotics. In recent years, plants have particularly played a pivotal role as viable sources for the discovery of strong antibacterial agents applied in the pharmaceutical, food, and animal feed industries. 

Plants produce various secondary metabolites that have antimicrobial properties such as phenolics, alkaloids, flavonoids, tannins, terpenoids, polyacetylenes, and quinones [6,11,12,13]. These phytochemical compounds, namely phytoalexins, are naturally occurring antimicrobials produced by plant as active defense mechanisms against phytopathogens such as bacteria, fungi, and viruses in the environment [14]. In addition, these biomolecules possess a wide spectrum of chemical properties including unique chemical scaffolds and complex structures, providing potentiating inhibitory effects associated with possible distinctive mechanisms of action against microorganisms [15,16]. There have been several previous studies reporting a significant advantage of these phytochemicals to impede the development of drug-resistant bacteria [17,18]. Some of these phytochemicals and plant extracts are designated by the United States Food and Drug Administration (FDA) as Generally Recognized As Safe (GRAS), and have low toxicity and good acceptability to food and medical applications [18]. All these features in combination support that plants are a promising source of natural antimicrobials.

There has been a growing interest in discovering new and effective antimicrobial substances from traditional Chinese medicinal plants (TCMP). In China, 11,118 species of plants have been used as medicinal materials for centuries [19]. By virtue of diverse bioactive compounds synthesized from the plants, their therapeutic applications have been reported in many ethnopharmacological studies including anticancer, hepatoprotection, anti-inflammatory, antidiarrheal antioxidant, antiviral, and antimicrobial activities [20,21]. Numerous studies have been demonstrated on the antimicrobial activities of TCMP extracts against different types of microbes, but there still remain challenges to discover novel antimicrobial molecules from TCMP. Therefore, 239 TCMP materials (89 families, 206 genera, and 233 species) were selected from the Chinese literature that have antibacterial effects in this present study. It was aimed to take a large-scale screening of TCMP for the assessment of potentially strong antibacterial activity against multidrug-resistant *S. aureus* to evaluate the provision of promising baseline information for the potential use of TCMP as novel antimicrobial agents in pharmaceutical, food and animal feed industries.

## 2. Results and Discussion

### 2.1. Screening of Antibacterial TCMP Extracts against Antibiotic-Resistant S. aureus

**Figure 1:**
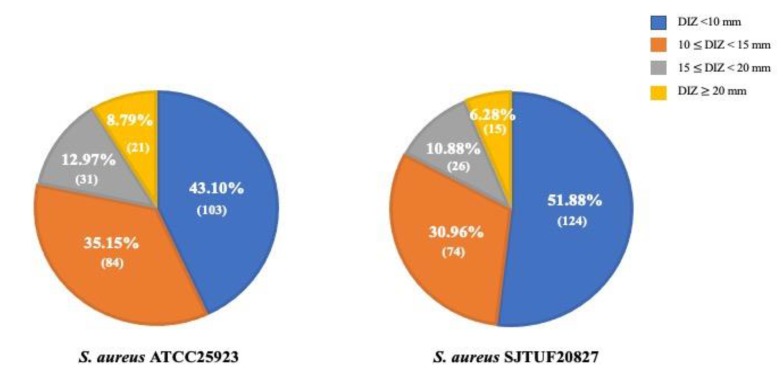
Screening of 239 TCMP extracts for antibacterial activities against *S. aureus* ATCC 25923 (reference strain) and SJTUF 20827 (antibiotic-resistant strain). Number in parentheses indicates the number of TCMP extracts, which exhibited DIZ value in four different ranges of DIZ value; DIZ < 10 mm, 10 ≤ DIZ < 15 mm, 15 ≤ DIZ < 20 mm and DIZ ≥ 20 mm.

The 239 ethanolic TCMP extracts were used to screen for their potential antibacterial activity against *S. aureus* including one reference strain ATCC 25923 and one antibiotic-resistant strain SJTUF 20827 based on the agar diffusion method (Table S1 in Supplementary Material and Figure 1). Antibacterial activities of TCMP extracts, evaluated by the measurement of diameter of inhibition zone (DIZ), were classified as very strong (DIZ ≥ 20 mm), strong (20 > DIZ ≥ 15 mm), moderate (15 > DIZ ≥ 10 mm), and weak (DIZ < 10 mm) [22]. Fifty-two out of 239 extracts showed strong antibacterial activities against *S. aureus* ATCC 25923, whereas 41 extracts had strong antibacterial activities against *S. aureus* SJTUF 20827 (DIZ ≥ 15 mm) that had similar values of DIZ (12.9–34.6 mm) with conventional antibiotics such as oxacillin (32 µg/mL) and ampicillin (4 µg/mL). Of the extracts, *Speranskia tuberculate* Baill. exhibited the best inhibitory effects on the growth of both reference and antibiotic-resistant *S. aureus* strains with DIZ values 26.8 and 25.2 mm, respectively. In this study, antibacterial properties of TCMP against *S. aureus* may be attributed to the simplicity of its cell membrane structure and composition in Gram-positive bacteria, which can be easily accessible to permeation of hydrophobic active compounds in TCMP [13]. Therefore, antibacterial molecules can directly access the target sites both on the cell wall and in the cytoplasm, resulting in pore formation, intercellular constituent leakages, structural or functional abnormalities of the bacterial membrane phospholipid bilayer, and inhibition of its biosynthesis [23,24,25,26]. In addition, these active compounds are commonly targeted at multiple sites rather than one specific site of antibacterial action [27,28]. For example, Ebelle et al. [29] reported that the methanolic extract of *Enantia chlorantha* had several modes of antibacterial action including the extension of the bacterial latency period, the deactivation of H+ - ATPases activity in cell membrane, the loss of the salt tolerance of the *S. aureus*, and inhibition of the biofilm formation. Since TCMP extracts contain diverse chemical substances, they probably influence the bacteria cell constituents or molecular targets by various mechanisms.

The results also revealed that the reference *S. aureus* was more susceptible to TCMP extracts compared with the antibiotic-resistant strain. These differences may be attributed to the fact that the antibiotic-resistant bacteria have acquired complex resistance mechanisms to survive in the presence of the antimicrobial molecules, rendering the antibacterial agents inactive. Indeed, bacterial pathogens have developed strategies for antibacterial resistance by chemical alteration and destruction of antibacterial molecules, decrease in their penetration, extrusion of the antibacterial compound, and interference in their access to target sites [30,31]. In addition, they are able to synthesize a protective polysaccharide layer on their surface, known as a bacterial biofilm [32]. These resistant factors might influence the activity of TCMP extracts against antibiotic-resistant *S. aureus* in this study. Further studies are needed to determine the mechanisms of antibacterial action of the extracts and their specific targets in antibiotic-resistant *S. aureus* strains.

### 2.2. Selected TCMP Extracts with a Wide Range of Antibacterial Activities against Multidrug-Resistant Bacteria

**Figure 2:**
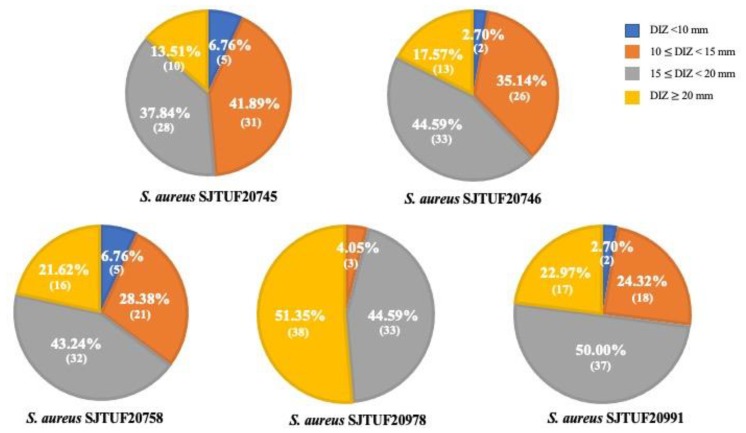
Selected TCMP extracts with the wide range of antibacterial activities against multidrug-resistant *S. aureus*. Number in parentheses indicates the number of TCMP extracts, which exhibited DIZ value in four different ranges of DIZ value; DIZ < 10 mm, 10 ≤ DIZ < 15 mm, 15 ≤ DIZ < 20 mm and DIZ ≥ 20 mm.

Based on initial screening results in which both the reference and antibiotic-resistant *S. aureus* model strains were found to be susceptible to TCMP extracts, we selected 74 strong TCMP extracts (DIZ ≥ 15 mm) and proceeded to the assessment of the wide range of antibacterial activities against multidrug-resistant *S. aureus* strains SJTUF 20745, 20746, 20758, 20978, and 20991 (Table 1). In this study, results showed that the selected TCMP extracts exhibited different antibacterial effects against multidrug-resistant pathogens; however, most extracts revealed strong and extensive antibacterial activities. As shown in Figure 2, 51.35%, 62.16%, 64.86%, 95.94%, and 77.97% of TCMP extracts possessed strong inhibitory effects (DIZ ≥ 15 mm) against *S. aureus* SJTUF 20745, 20746, 20758, 20978, and 20991, respectively. 

However, certain TCMP extracts such as *Cnidium monnieri* Cusson and *Nervilia fordii* Schltr. showed poor inhibitory effects against most multidrug-resistant *S. aureus* strains (Table 1). In total, TCMP extracts of *S. tuberculate*, *Acacia catechu* (L.f.) Willd., *Coptis chinensis* Franch., *Quercus infectoria* Oliv., *Leontice kiangnanensis* P.L. Chiu, *Rhus chinensis* Mill., *Rabdosia rubescens* (Hemsl.) H. Hara, and *Dalbergia odorifera* T.C. Chen had the best antibacterial activities against antibiotic-resistant bacteria with DIZ values ranging from 18.2 to 31.9 mm compared with tested conventional antibiotics (DIZ = 11.9–21.7 mm). The strong antibacterial activities of these TCMP have been reported in many studies, which are correlated well with the results obtained in this study. For example, previous study showed that the methanolic extract of *A. catechu* at a concentration of 100 mg/mL had a strong antibacterial activity against *S. aureus*, with DIZ value 20 mm [33]. Feng and Xu [34] demonstrated that *R. rubescens* extract had broad-spectrum inhibitory effects on *S. aureus*, *Symphoricarpos albus* and *Bacillus subtilis* strains with DIZ values in range of 17.5–22.8 mm, 18.2–22.3 mm, and 17.3–25.6 mm, respectively. The antibacterial effects of *Q. infectoria* gall extract against multidrug-resistant bacteria has also been studied [35]. The ethanolic extracts of *Q. infectoria* (1 mg/disc) exhibited inhibitory effects against methicillin-resistant *S. aureus* (MRSA, DIZ = 13.3–15.3 mm), methicillin-resistant coagulase-negative *Staphylococcus* (MRCoNS, DIZ = 19.3 mm), and multidrug-resistant *Acinetobacter* spp. (DIZ = 12.7 mm).

### 2.3. Minimum Inhibitory Concentration (MIC) and Minimum Bactericide Concentration (MBC) of TCMP Extracts against Multidrug-Resistant S. aureus

Next, 18 selected TCMP extracts with the highest DIZ values were subjected to investigations of their MIC and MBC against multidrug-resistant *S. aureus* strains (Table 2). The MIC values for the extracts ranged from 0.1 to 12.5 mg/mL and the MBC values ranged from 0.78 to 25 mg/mL. Of the TCMP extracts evaluated, multidrug-resistant *S. aureus* strains were impacted with a high degree of susceptibilities, expressed as MIC, to *R. chinensis* (0.1–0.195 mg/mL), followed by *Q. infectoria* (0.195 mg/mL), *C. chinensis* (0.195–0.39 mg/mL), *D. odorifera* ( 0.39 mg/mL), *Agrimonia pilosa* Ledeb. (0.1–0.78 mg/mL), *A. catechu* (0.195–0.78 mg/mL), *Phellodendron chinense* C.K. Schneid. (0.195–0.78 mg/mL), *Terminallia chebula* Retz. (0.39–0.78 mg/mL), *Oroxylum indicum* Vent. (0.39–1.56 mg/mL), and *Spatholobus suberectus* Dunn. (0.39–1.56 mg/mL). Conversely, *Isatis tinctoria* L. showed relatively poor antibacterial activity with the high MIC values (3.125–12.5 mg/mL) and MBC values (12.5–25 mg/mL). Accordant results were previously reported from other studies. In one example, Tian et al. [36] reported that gall extract of *R. chinensis* was more effective against *S. aureus* (MIC = 0.25 mg/mL) among tested microorganisms. Moirangthem et al. [37] found that the bark extract from *O. indicum* exhibited strong antibacterial effects against *S. aureus* (MIC = 62.5 µg/disc), and also showed a broad antibacterial spectrum against Gram-positive and Gram-negative bacteria with the MIC values ranging from 62.5 to 250 µg/disc. Similar to the results obtained in this study, Tayel et al. [38] reported that the extract of *Q. infectoria* exhibited strong inhibitory effects against *S. aureus* with the MIC value for 0.313 mg/mL. Wan et al. [35] also determined the antibacterial activities of *Q. infectoria* that MIC values of the ethanolic extract against multidrug-resistant bacteria were in the range of 0.03 to 0.63 mg/mL, indicating their strong antibacterial activities. Other TCMP extracts with good antibacterial activities against other bacterial pathogens reported in other studies [39,40,41], are in agreement with the results in Table 2. The observed slight differences in the susceptibility of test bacteria were due to different solvents, extraction methods, antibacterial test methods, harvesting time, and the variation in the proportion of bioactive compounds.

In general, this study selected out some TCMP with strong and extensive growth inhibitory effects on multidrug-resistant *S. aureus*, including *R. chinensis*, *Q. infectoria*, *C. chinensis*, *D. odorifera*, *A. pilosa*, *A. catechu*, *P. chinense*, *T. chebula*, *O. indicum*, *S. suberectus*, *S. tuberculate*, *Inula japonica* Thunb., *R. rubescens*, and *Salvia miltiorrhiza* Bunge. Additionally, to the best of our knowledge, our study is the first to report the promising antimicrobials of plant extracts, including *I. rotunda*, *I. japonica*, *L. kiangnanensis*, *S. tuberculate* and *F. fallax*, all of which had strong and broad-range of antibacterial activities against multidurg- resistant *S. aureus*.

### 2.4. Total Phenolic Content (TPC) and Total Flavonoid Content (TFC) of TCMP Extracts

Plant species naturally produce various secondary metabolites leading to possession of valuable biological activities including antibacterial activity [20]. Selecting a potential antibacterial agent from plant-derived compounds might be an appropriate strategy since these substances are produced by the outcome of plant defense responses against biotic stress from bacterial, fungi, viruses, and abiotic stress, and commonly have antibacterial activities [18]. Major plant-derived compounds that are responsible for antibacterial activity include phenolics, phenolic acids, flavonoids, quinones, tannins, coumarins, terpenoids, and alkaloids [16]. The structural diversity and various chemical compositions of antibacterial compounds result in strong and broad-range of antibacterial activities with diverse mechanisms of antibacterial action [15]. 

Multiple studies have reported that phenolic compounds (phenolic acids, flavonoids, quinones, coumarins, lignans, stilbenes, and tannins) presented in TCMP contribute to good antibacterial properties against bacterial pathogens [42,43,44]. Polyphenolic compounds such as flavan-3-ols, flavonols, tannins, and phenolic acids are known to exhibit wide spectra and strong antimicrobial activity compared with other polyphenols [45]. The phenolic compounds are also able to suppress microbial virulence factors such as quorum sensing, bacterial biofilms, bacterial motility, bacterial toxins, and bacterial surfactant [16]. For example, Wang et al. [46] reported that vesticarpan derived from *D. odorifera* as the phenolic compound showed antibacterial activity against *Ralstonia solanacearum*. Four flavonoids isolated from *D. odorifera*, including sativanone, (3R)-vestitone, liquiritigenin and isoliquiritigenin, exhibited strong antibacterial activity against *R. solanacearum* with the DIZ values ranging from 11.2 to 16.6 mm [47]. Sithisarn et al. [48] demonstrated that flavones such as baicalein, baicalin, and chrysin in *O. indicum* are active compounds for antibacterial activities against *Staphylococcus intermedius*, *Streptococcus suis*, *Pseudomonas aeruginosa* and extended-spectrum *β*-lactamase (ESBL)-producing *Escherichia coli*. Cho et al. [49] also found active flavonoids like 7-hydroxy-6-methoxy-flavanone and formononetin isolated from *S. suberectus* that inhibited *S. aureus* derived sortase A, which is responsible for anchoring surface protein virulence factors.

Therefore, we further determined the phenolic content in 18 TCMP extracts with strong antibacterial effect to demonstrate whether their major antibacterial compounds are attributed to polyphenols. Total phenolic content (TPC) and total flavonoid content (TFC) were determined by the Folin-Ciocalteu method and AlCl_3_-based colorimetric method, respectively. The TPC in the extracts were in the range of 31.4 to 646 mg gallic acid equivalent (GAE)/g dry weight (DW), whereas TFC were in the range of 5.27 to 377 mg catechin equivalent (CE)/g DW (Table 3). Consistent with previous studies, strong antibacterial TCMP extracts such as *R. chinensis*, *Q. infectoria*, *A. Pilosa*, *A. catechu* and *S. suberectus* contained high amounts of polyphenols, with TPC 632, 646, 371, 545, and 489 mg GAE/g DW, respectively, leading to a suspicion that polyphenols might be the actual active compounds responsible for antibacterial activity against *S. aureus*. A case in point being the gall extract of *Q. infectoria* which presented the highest polyphenol level (646 mg GAE/g DW), but it presented a relatively low flavonoids level (38 mg CE/g DW), indicating that major phenolic components in *Q. infectoria* might be consisted of non-flavonoids such as phenolic acids, stilbenes and lignans. Arina and Harisun [50] found that *Q. infectoria* gall mainly contained polyphenols with a high concentration of tannic acid (2233 mg/g). Tannins, well-known as antibacterial agents, have been shown to inhibit the growth of various pathogens by destroying their bacterial plasma membrane and forming hydrogen bonds between tannins and the proteins in bacterial cells, resulting in the protein denaturation and their coagulation [51]. Conversely, it was also found that some other strong antibacterial TCMP extracts like *C. chinensis*, *S. tuberculate*, and *S. miltiorrhiza* included relatively low TPC (95, 31.4 and 56 mg GAE/g DW, respectively) and TFC (23.1, 13.4 and 34.5 mg CE/g DW, respectively), suggesting that their major antibacterial substances might be nonphenolic antibacterial compounds. 

Although the spectrophotometric methods such as Folin-Ciocalteu method and AlCl_3_-based colorimetric method are commonly used to quantify total phenolics in plant extracts [52,53,54,55,56,57], these analyses are not accurate measurements. The Folin-Ciocalteu assay can contribute to the overestimation of TPC due to the presence of reducing compounds such as ascorbic acid, reducing sugars, and aromatic amino acids (tyrosine and tryptophan), leading to the disruption of phenolic oxidation reaction [58,59,60,61]. Similar to the limitation for TPC, the AlCl_3_-based method for TFC has a constraint on the measurement of all classes of flavonoids in the extracts and the considerable contents of total flavonoid can be attributed to the presence of phenolic acids in the extract during the absorbance measurement at 510 nm [62]. Therefore, more accurate and precise analyses such as chromatographic methods would be necessary to conduct for qualification of total phenolic compounds in the TCMP extracts. In this present study, these spectrophotometric methods were used for simple comparison among the selected TCMP extracts. However, further research would be required using more accurate and reliable analytical methods such as chromatographic assays in order to investigate phytochemical constituents and to identify major antimicrobial compounds in each extract.

### 2.5. Cytotoxicity and Safety of the TCMP Extracts

**Figure 3:**
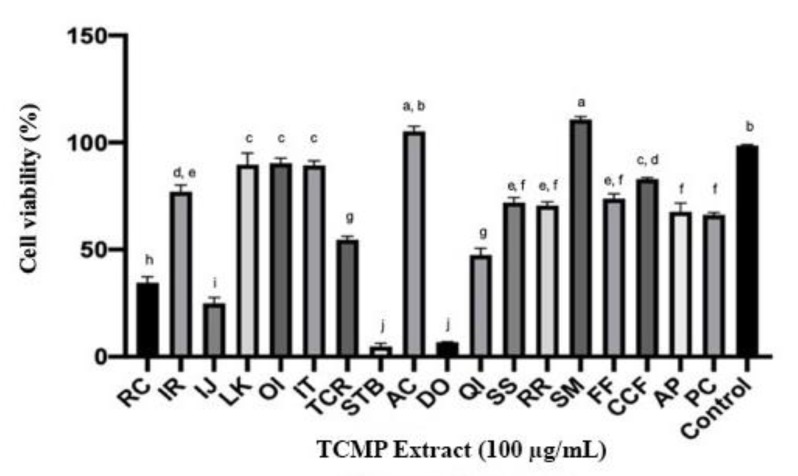
The viability of HFF cells exposed to selected TCMP extracts (100 µg/mL) for 24 h assessed by the colorimetric assay using MTT. RC = *R. chinensis*; IR = *I. rotunda*; IJ = *I. japonica*; LK = *L. kiangnanensis*; OI = *O. indicum*; IT = *I. tinctorial*; TCR = *T. chebula*; STB = *S. tuberculate*; AC = *A. catechu*; DO = *D. odorifera*; QI = *Q. infectoria*; SS = *S. suberectus*; RR = *R. rubescens*; SM = *S. miltiorrhiza*; FF = *F. fallax*; CCF = *C. chinensis*; AP = *A. Pilosa*; PC = *P. chinense;* Control = 0.1% DMSO. Results are expressed as mean ± standard deviation (SD) and the experiments were conducted in triplicate (*p* < 0.05).

In order to utilize the crude TCMP extracts as antimicrobial agents in pharmaceuticals, food, and animal feed industries, it is essential to ensure their safety. The 18 TCMP extracts with good antibacterial effect were evaluated for their cytotoxicity in normal human foreskin fibroblast (HFF) cells. Cell viability varies with the extracts when the cells were exposed at the concentration of 100 μg/mL for 24 h (Figure 3). *S. tuberculate* extract had a considerably low level of cell viability with 4.83%, followed by *D. odorifera* (7.14%), *I. japonica* (24.9%), *R. chinensis* (34.5%), and *Q. infectoria* (47.5%). Additionally, in order to investigate the cytotoxic effects of the TCMP extracts, dose-response experiments were conducted (Figure S1 in Supplementary Material) and the median lethal concentration (LC_50_) values were calculated (Table 4). The cytotoxicity of plant extracts with LC_50_ value ≤ 20 μg/mL was regarded as a possible cytotoxic plant extract [63]. The results indicated that most plant extracts had low cytotoxicities with considerably high LC_50_ value ≥ 100 µg/mL, excluding *S. tuberculate* (LC_50_ = 25.9 µg/mL), *D. odorifera* (LC_50_ = 44.1 µg/mL), *I. japonica* (LC_50_ = 54.1 µg/mL), *R. chinensis* (LC_50_ = 77.6 µg/mL), and *Q. infectoria* (LC_50_ = 91.6 µg/mL), supporting by previous studies that also found a weak cytotoxicity of *T. chebula* [64,65], *S. suberectus* [66], *Q. infectoria* [67,68] and *O. indicum* [37]. However, it is necessary to test the toxicity by in vivo studies, since in vitro cellular toxicity might provoke different consequences in animals associated with gut interactions and bioavailability of the extracts [18]. 

To verify the safety of plant extracts, selectivity index (SI) of plant extracts was also determined by LC_50_ dividing by MIC value (Table 4). The SI value greater than 1 indicates that a plant extract is more toxic to the pathogen than the host cell [63]. In other words, the plant is safe and possible to be developed as antibacterial agents [13]. *I. japonica* extract had the lowest SI value of 0.02 against *S. aureus*, followed by *S. tuberculate* (0.03), *D. odorifera* (0.11), *Q. infectoria* (0.47), and *R. chinensis* (0.77). Besides the five extracts, most other plant extracts had strong inhibitory effects on *S. aureus*, but were less cytotoxic to human cells, suggesting that these plants could be developed as herbal medicine, food additive or preservative in the future. 

### 2.6. Correlations Analysis among Polyphenolic Content, Antibacterial Effect, and Cytotoxicity of TCMP Extracts Cytotoxicity and Safety of the TCMP Extracts

The presence of polyphenolic compounds in the TCMP extracts can be related to strong antibacterial activity against multidrug-resistant *S. aureus*. In previous study, Shan et al. [42] demonstrated good linear correlations of phenolic content with the antibacterial activity of medicinal herbs against foodborne bacteria including *S. aureus* (*r*^2^ = 0.93). Pavić et al. [52] also reported that TPC in a medicinal plant, namely *Ruta graveolen* L., was strongly correlated to the MIC values of *E. coli*, *B. subtilis*, and *S. aureus* (*r* = 0.973, *p* < 0.050). These results suggest that polyphenols might contribute to antibacterial properties of plant extracts. The antibacterial activity of polyphenols can involve various mechanisms of action such as disrupting the cell integrity, destroying membrane proteins, increasing permeability of cell membrane, inhibiting biofilm formation, inactivating microbial enzymes, up/down-regulating proteins involved in DNA and RNA synthesis, and deprivation of metal iron by their chelating ability [69,70]. For instance, some phenolics such as chlorogenic acid, tannic acid, and caffeic acid inhibited on the bacterial growths due to hyperacidification at the plasma membrane interface, resulting in disrupting H^+^-ATPase pump and thereby causing cell death [53]. 

**Figure 4:**
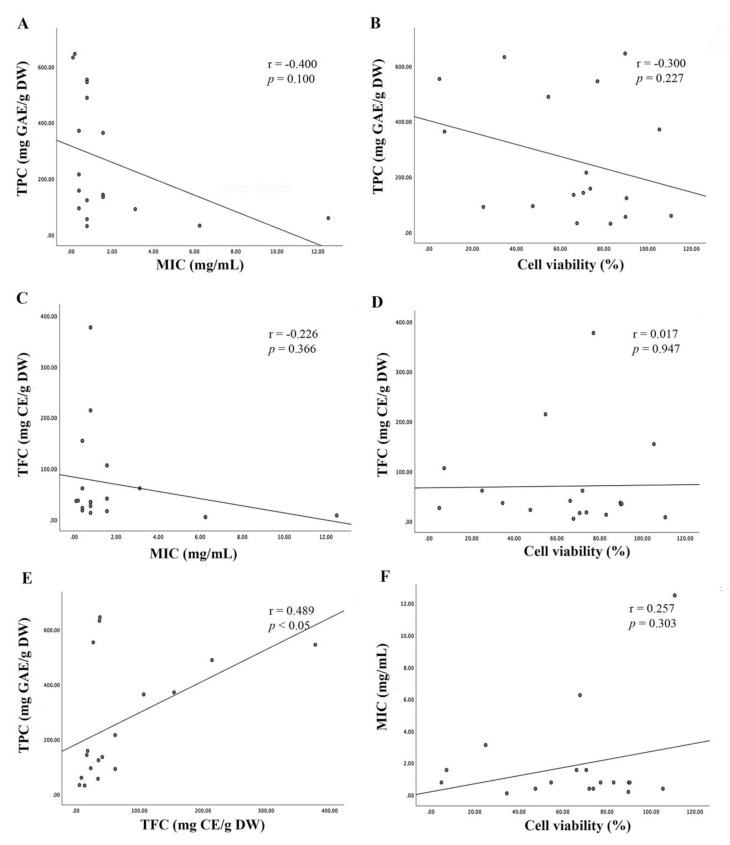
Pearson correlation analysis. (**A**) Correlation of total phenolic content (TPC) with minimum inhibitory concentration (MIC). (**B**) Correlation of TPC with cell viability (%) at 100 µg/mL. (**C**) Correlation of total flavonoid content (TFC) with MIC. (**D**) Correlation of TFC with cell viability (%). (**E**) Correlation of TPC with TFC. (**F**) Correlation of MIC with cell viability (%).

In order to understand the interrelationships between the antibacterial activity of TCMP extracts and polyphenolic contents, 18 TCMP extracts with good effects were used in an analysis of correlations among MIC, TPC, and TFC values (Figure 4). The results with high *p* values (*p* > 0.05) are interpreted as without statistical significance that might be confounded by the small sample size (*n* = 18) in this study because of *p* value’s dependence on the sample size [71,72]. Unlike *p* value, the effect size is independent of sample size [71]. In this study, Pearson’s correlation coefficient (r) was introduced as an effect size index to quantify difference among variables and to understand the correlations of antibacterial activity with phenolic contents. A moderate negative correlation was obtained between TPC and MIC with Pearson’s correlation coefficient r = −0.400 (*p* = 0.100), and a very weak correlation was also shown between TFC and MIC with r = −0.226 (*p* = 0.366), in agreement of the results obtained by a previous study [54], showing moderate correlations of antibacterial activity with phenolic contents in spice extracts (r = 0.541, *p* < 0.001). 

The r values were low and moderate based on the standards of effect size, which are classified as small (r = ± 0.2), medium (r = ± 0.3), and large (r = ± 0.5) [71]. The effect size can be influenced by factors such as amount of variability, linear relationship among two variables, presence of outlier and measurement errors [73]. The relationships between phenolic contents and antibacterial activities showed the inclination to nonlinearity, leading to the low values of Pearson’s correlation (Figure 4). In addition, the narrow range of MIC values (0.01–12.5 mg/mL) compared to TPC (31.4–646 mg GAE/g DW) and TFC (5.27–214 mg CE/g DW) might be attributed to low r values. This tendency for the weak correlations between phenolic contents and antibacterial activity might be suspected to the high levels of TPC, resulting from overestimated TPC values due to the presence of reducing compounds in the TCMP extracts [58,59,60,61]. Therefore, the phytochemical profiles of TCMP extracts need to be investigated by accurate and precise analyses in the future to reliably comprehend the correlations of antibacterial activity with phenolic compounds in the extracts. In other respects, the results may be also suggested that antibacterial activity of TCMP extracts is attributed to nonphenolic substances, especially the extracts of *C. chinensis*, *S. tuberculate*, and *S. miltiorrhiza*, which had relatively low polyphenols contents (Table 3). Indeed, Lee et al. [74] discovered that a terpenoid compound, namely dihydrotanshinone I, isolated from the roots of *S. miltiorrhiza* was the strong antibacterial compound against the broad range of gram-positive bacteria such as *B. subtilis* with a low MIC value of 3.1 µg/mL.

For the purpose of evaluating possible antibacterial compounds in TCMP extracts with their safety for use, we also analyzed the correlations of antibacterial activity with cell viability (%), showing the weak positive correlation with r = 0.257 (*p* = 0.303) (Figure 4). The results indicate that cytotoxic compounds might be discordant with antibacterial compounds [13,63]. Indeed, Dzoyem et al. [75] found that antibacterial compounds such as ursolic acid, quercitrin, and entadanin from *Entada abyssinica* were relatively low cytotoxic on Vero monkey kidney cells. Two flavonoids isolated from *Pappea capensis*, quercetin-3-*O*-rhamnoside and epicatechin, exhibited a wide range of antibacterial activity against *B. subtilis*, *S. aureus*, *E. coli* and *Klebsiella pneumoniae* and showed considerably low cytotoxic on Vero monkey kidney cells with LC_50_ values > 200 μg/mL [76]. Therefore, there still remain the possibilities to identify strong antibacterial compound(s) from *S. tuberculate*, *I. japonica*, *D. odorifera* and *Q. infectoria* that were relatively more toxic to the pathogen than the host cells (Table 4).

## 3. Materials and Methods

### 3.1. Chemicals and Reagents

Dimethyl sulfoxide (DMSO) and 3-(4,5)-dimethylthiazol-2-yl)-2,5-diphenyltetrazolium bromide (MTT) were purchased from Beyotime (Shanghai, China). Ethanol was purchased from Titan Chem. (Shanghai, China). Luria Bertani (LB) broth, agar bacteriological, and Mueller–Hinton broth were from Oxiod (Basingstoke, UK). Ampicillin and oxacillin were purchased from Meilune (Dalian, China). Sodium chloride, sodium nitrite, sodium hydroxide, potassium chloride, disodium phosphate and monopotassium phosphate were purchased from General-Reagent^®^ (Shanghai, China). Aluminum chloride was purchased from Sinopharm Chemical Reagent (Shanghai, China). Sodium carbonate was from J&K Scientific (Beijing, China). Resazurin was purchased from Adamas (Shanghai, China). Gallic acid was from 87 Energy Chemical (Shanghai, China). Folin-Ciocalteu reagent was from Macklin (Shanghai, China). Deionized water was used in all experiments.

### 3.2. Collection of Plant Samples

The 239 dried TCMP (89 families, 206 genera, and 233 species) were collected from the markets in Shanghai, China. The TCMP samples presented in Table 1 and Table S1 in the Supplementary Material were sorted in term of family, scientific name, and common name identified from GBIF (http://www.gbif.org/) and Tropicos^®^ (http://www.tropicos.org/).

### 3.3. Preparation of Plant Extracts 

The dried TCMP samples were pulverized by a lab-scale miller (S025, IKA, Staufen, Germany). In this study, ethanol was used as an extraction solvent due to its safety and good extraction activity to obtain the desired antimicrobial components from the plant materials [77]. 4.0 g dried powder of each TCMP was extracted two times with 40 mL of 80% (*v*/*v*) ethanol using an ultrasound-assisted extraction method (1 h, 40 °C, and 480 W) to improve extraction efficiency and the yield of antibacterial compounds in the plants [55,78,79]. Each extract was centrifuged at room temperature (900× *g*, 15 min), and the supernatants were collected, combined, and concentrated using a rotary evaporator (RE-52AA, Shanghai Ya Rong Co., Ltd, Shanghai, China) at 40 °C under vacuum. The concentrated extract was dried by a vacuum freeze-dryer (SJIA-5FE, Ningbo Shuang Jia instrument Co., Ltd, Ningbo, China). The freeze-dried extract was dissolved in dimethyl sulfoxide (DMSO) at 100 mg/mL and was stored at −20 °C for further use. 

### 3.4. Microorganisms and Culture Samples

One reference strain of *S. aureus* ATCC 25923 and six antibiotic-resistant *S. aureus* strains were used in this study. The list of six *S. aureus* consisted of five multidrug-resistant strains (SJTUF 20745, SJTUF 20746, SJTUF 20758, SJTUF 20978, and SJTUF 20991), and one erythromycin-resistant strain (SJTUF 20827), verified in our previos study [54], is shown in Table 5. *S. aureus* ATCC 25923 was used as a reference strain. Single colonies of the bacteria growing on Luria Bertani (LB) agar plate were inoculated in LB culture medium and grown overnight in a shaking incubator at 37 °C and 250 rpm. The bacterial suspension was used at a concentration of 1 × 10^6^ colony-forming units (CFU)/mL for the following antibacterial experiments.

### 3.5. Determination of Antibacterial Activity

#### 3.5.1. Measurement of Diameter of Inhibition Zone (DIZ)

The antibacterial activity of 239 TCMP extracts (100 mg/mL) against *S. aureus* ATCC 25923 and SJTUF 20827 was evaluated based on the DIZ determined by agar diffusion methods as described by Chan et al. [22] with some modifications. Ampicillin (32 μg/mL) and oxacillin (4 μg/mL) were used as the positive controls, and DMSO (60 μL/cup) were used as the negative controls. The DIZ values less than or equal to 8.0 mm was regarded as no antibacterial activity. In order to test the wide-spectrum antibacterial effects on multidrug-resistant *S. aureus*, 74 TCMP extracts with DIZ ≥ 15 mm from the screening results were further investigated their DIZ against another five multidrug-resistant *S. aureus* strains (SJTUF 20745, SJTUF 20746, SJTUF 20758, SJTUF 20978, and SJTUF 20991) as the procedure above. The assay was conducted in triplicate in two independent experiments.

#### 3.5.2. Determination of Minimum Inhibitory Concentration (MIC) and Minimum Bactericide Concentration (MBC)

TCMP samples with DIZ ≥ 20 mm were used to determine their MIC and MBC against *S. aureus* strains in triplicate. The MIC and MBC were determined by a serial dilution microplate method and Mueller–Hinton (MH) agar counting, respectively [22,80]. Ampicillin and oxacillin were used as the positive controls. This assay was performed in triplicate in three independent experiments.

### 3.6. Determination of the Total Phenolic and Flavonoid Content 

Total phenolic content (TPC) was determined using a Folin-Ciocalteu method, as previously described by Blainski et al. [81]. To quantify TPC, a linear calibration curve of gallic acid was established as a standard ($y\;=\;0.0511+\;9.014x,r^{2}=\;0.9916$). TPC was calculated using the linear equation and expressed as mg gallic acid equivalent (mg GAE)/g dry weight (DW) of the sample. Total flavonoids content (TFC) was quantified using an AlCl_3_-based colorimetric method [56]. Catechin was used as a standard to establish a linear calibration curve with function ($y\;=\;0.0229\;+\;0.0033x,r^{2}=\;0.999$). The assay was performed in triplicate in two independent experiments. TFC was calculated using the linear equation and expressed as mg catechin equivalent (mg CE)/g DW of sample.

### 3.7. In vitro Cytotoxicity Assay 

The cytotoxicity of 18 TCMP ethanolic extracts on human foreskin fibroblast (HFF) cells, which are human normal cells, was determined by the colorimetric assay using 3-(4,5)-dimethylthiazol-2-yl)-2,5-diphenyltetrazolium bromide (MTT) as described by Senthilraja and Kathiresan [82] with slight modification. Briefly, $2\times 10^{4}$ cells/mL were cultured with Dulbecco’s modified Eagle’s medium (DMEM) supplemented with 10% fetal bovine serum into each well of 96-well microplates and incubated at 37 °C in a humidified 5% CO_2_ incubator overnight. The cells were treated with various concentrations (ranging from 1.56 to 100 µg/mL) of TCMP extracts and incubated at 37 °C for 24 h, whereas the untreated cells were used as control. Next, cells in each well were treated with 100 µL MTT (5 mg/mL, in PBS) and were maintained for 3 h at 37 °C in the dark. After removal of MTT solution, 100 µL DMSO was added to dissolve insoluble formazan crystal. The absorbance was measured at 570 nm by a microplate reader (SpectraMax iD3, Molecular Devices, Silicon Valley, NC, USA). This assay was conducted in triplicate in four independent experiments. The percentage of cell viability was calculated based on the following equation:

(1) $$\mathrm{Cell}\;\mathrm{viability}\;\left(\%\right)=({\mathrm{Absorbance}}_{\mathrm{sample}}/{\mathrm{Absorbance}}_{\mathrm{control}})\times 100,$$

The median lethal concentration (LC_50_) was calculated according to the log dose-response curve of cytotoxicity against concentration and was defined as a 50% reduction of cell viability compared with the control. The selectivity index (SI) value was calculated based on the equation below [63]:

(2) $$\mathrm{Selective}\;\mathrm{index}\;\left(\mathrm{SI}\right)={\mathrm{LC}}_{50}\;\left(\mathrm{mg}/\mathrm{mL}\right)/\mathrm{MIC}\;(\mathrm{mg}/\mathrm{mL}),$$

### 3.8. Statistical Analysis

All assays were conducted in triplicate and all experimental results were expressed as mean ± standard deviation (SD). Statistical analysis was performed using Microsoft Excel 2018 (Microsoft, Seattle, WA, USA) and SPSS 22.0 (IBM SPSS Statistics, IBM Corp, Somers, NY, USA). The cytotoxicity data (cell viability and LC_50_) were analyzed by GraphPad Prism 8 (GraphPad Software, San Diego, CA, USA). Statistical significance was defined at *p*-value less than 0.05.

## 4. Conclusions

This study conducted the large-scale screening of 239 TCMP extracts to discover strong antibacterial activities against multidrug-resistant *S. aureus*. This study selected out several TCMP extracts with promising antibacterial activity as well as low cytotoxicity, including *R. chinensis*, *I. rotunda*, *L. kiangnanensis*, *O. indicum*, *I. tinctoria*, *T. chebula*, *A. catechu*, *S. suberectus*, *R. rubescens*, *S. miltiorrhiza*, *F. fallax*, *C. chinensis*, *A. pilosa*, and *P. chinense*. The results of plant extracts, *I. rotunda*, *I. japonica*, *L. kiangnanensis*, *S. tuberculate* and *F. fallax*, were first reported about their antibacterial effects on *S. aureus* in the present study. Further study would be required to investigate phytochemical profiles in TCMP extracts by more sensitive analytical methods. It would be necessary to identify their antibacterial active compounds, potential mechanisms of antibacterial action, and in vivo toxicity prior to applying them as new antibacterial agents in pharmaceutical, food, and animal feed industries.

## Figures and Tables

**Table 1:** Antibacterial activities of 74 selected TCMP extracts against multidrug-resistant *S. aureus* strains based on diameter of inhibition zone (DIZ).

No. ^1^	Family	Scientific Name	Common Name	Extracted Plant Part	DIZ (mm) against *S. aureus*				
					SJTUF 20745	SJTUF 20746	SJTUF 20758	SJTUF 20978	SJTUF 20991
3	Anacardiaceae	*Rhus chinensis* Mill.	Nutgall tree	gall	21 ± 1	21.5 ± 0.1	21.8 ± 0.4	25 ± 0.6	20.5 ± 0.3
8	Apiaceae	*Cnidium monnieri* Cusson	Monnier’s snowparsley	fruit	9	12.6	NIZ	17.6 ± 0.1	10.5 ± 0.2
22	Aquifoliaceae	*Ilex rotunda* Thunb.	Kurogane holly	bark	17.2 ± 0.5	17.6 ± 0.6	19.9 ± 0.8	17.4 ± 0.2	18.3
26	Araceae	*Spirodela polyrrhiza* (L.) Schleid.	Common duckweed	aerial part	9.1	10.4 ± 0.1	10.3 ± 0.1	17.5 ± 0.1	11.9
31	Aristolochiaceae	*Asarum heterotropoides* F. Schmidt	Manchurian wildginge	rhizome & root	14.3 ± 0.2	18	15.2 ± 0.2	29.8 ± 0.2	14.1 ± 0.1
36	Asphodelaceae	*Aloe ferox* Mill.	Cape aloe	dried gel	8.3	11.6 ± 0.4	8.7	14.6 ± 0.5	10.2 ± 0.3
46	Asteraceae	*Elephantopus scaber* L.	Cucha cara	aerial part	12.3 ± 0.5	13.2 ± 0.1	13.2 ± 0.2	15.1	13.8 ± 0.6
49		*Hemistepta lyrata* Bunge	Lyre-shape hemistepta	aerial part	12.4	12.3 ± 0.3	12.1 ± 0.4	16	14.3
51		*Inula japonica* Thunb.	Inula flower	flower	16.4 ± 0.4	17 ± 1	15.7	20 ± 0.5	20.3 ± 0.1
58	Berberidaceae	*Leontice kiangnanensis* P. L. Chiu	Rhizoma corydalis	tuberous root	19.1 ± 0.7	23.7 ± 0.3	22 ± 1	25.6 ± 0.1	22.4 ± 0.4
61	Bignoniaceae	*Oroxylum indicum* Vent.	Indian trumpet flower	seed	18.5 ± 0.3	25 ± 1	24.2 ± 0.2	30 ± 1	22.4 ± 0.2
62	Boraginaceae	*Lithospermum erythrorhizon* Siebold & Zucc.	Purple gromwell	leaf	11.8 ± 0.2	16.3 ± 0.2	13.5 ± 0.1	15.3 ± 0.1	13.4
65	Brassicaceae	*Isatis tinctoria* L.	Dyer’s woad	leaf	21 ± 1	22 ± 1	23 ± 1	24.8 ± 0.7	16.9 ± 0.3
81	Combretaceae	*Terminalia chebula* Retz.	Myrobalan	fruit	16.8 ± 0.3	18.1	20 ± 0.2	24.5 ± 0.1	20.2 ± 0.1
87	Dioscoreaceae	*Dioscorea bulbifera* L.	aerial yam	tuberous root	21 ± 1	21 ± 1	19.2	24.6 ± 0.5	20.8 ± 0.1
90	Ebenaceae	*Diospyros kaki* Thunb.	Chinese persimmon	calyx	16.7 ± 0.4	16.2 ± 0.3	16.7	19.5 ± 0.1	17.2 ± 0.2
92	Ericaceae	*Pyrola calliantha* Andres	Chinese pyrola	aerial part	13.7 ± 0.1	21.3 ± 0.5	13.3	18.2 ± 0.2	15.4 ± 0.1
95	Euphorbiaceae	*Euphorbia humifusa* Willd.	Herba euphorbiae humifusae	aerial part	13.8 ± 0.2	13	16.2 ± 0.6	18.1 ± 0.4	17.7 ± 0.2
96		*Speranskia tuberculata* Baill.	Herba speranskiae tuberculatae	aerial part	24.1 ± 0.4	25 ± 2	22.8 ± 0.3	27.7 ± 0.4	27.1 ± 0.5
97	Fabaceae	*Acacia catechu* (L.f.) Willd.	Catechu	branch	23 ± 0.8	25 ± 2	27 ± 1	32 ± 2	25.1 ± 0.3
101		*Cassia occidentalis* L.	Coffee senna	seed	14.9 ± 0.1	14.1 ± 0.1	13.8 ± 0.4	14.8 ± 0.2	10.3 ± 0.1
102		*Cassia tora* L.	Sickle Senna	seed	14 ± 0.4	14.2 ± 0.4	13.2 ± 0.8	16.6 ± 0.5	15.9 ± 0.1
103		*Dalbergia odorifera* T. C. Chen	Fragrant rosewood	trunk	18.2 ± 0.5	20 ± 1	20 ± 1	28 ± 1	21.4 ± 0.5
107		*Gleditsia sinensis* Lam.	Chinese honey locust	branch	14.6 ± 0.4	15.8 ± 0.2	17.4	20 ± 1	15.2 ± 0.4
109		*Glycyrrhiza uralensis* Fisch.	Licorice	rhizome & root	12.8 ± 0.1	13.7 ± 0.4	16.1 ± 0.2	17.4	14.8 ± 0.1
110		*Lablab purpureus* (L.) Sweet	Lablab Bean	seed	14.6	16.8 ± 0.3	19.2	22 ± 1	17.5
112		*Quercus infectoria* Oliv.	Aleppo oak	gall	22.4 ± 0.5	22.3 ± 0.4	24.8 ± 0.5	26.6 ± 0.1	22.8
114		*Sophora tonkinensis* Gagnepain	Vietnamese sophora	rhizome & root	14.3 ± 0.4	15.3 ± 0.2	13.5	20.2 ± 0.1	15.8
115		*Spatholobus suberectus* Dunn	Caulis spatholobi	stem	17 ± 0.1	17.2 ± 0.2	19 ± 1	20.8 ± 0.2	19.4 ± 0.2
122	Hypericaceae	*Hypericum japonicum* Thunb.	Matted St. John’s-wort	aerial part	17.8 ± 0.4	17.1 ± 0.1	14.7	22 ± 1	18.9 ± 0.2
126	Lamiaceae	*Isodon serra* Kudo	Herba rabdosiae	aerial part	15.8 ± 0.2	15.4 ± 0.2	18.7 ± 0.1	21.7 ± 0.1	17.3 ± 0.3
132		*Rabdosia rubescens* (Hemsl.) H. Hara	Blushred rabdosia	aerial part	18.8 ± 0.3	23 ± 1	19 ± 1	23.5 ± 0.1	22 ± 1
133		*Salvia miltiorrhiza* Bunge	Chinese salvia	rhizome & root	17.3 ± 0.5	18.9 ± 0.5	22.3 ± 0.3	19.1 ± 0.4	19 ± 0.3
139	Lardizabalaceae	*Sargentodoxa cuneata* Rehder & E. H. Wilson	Sargentgloryvine	stem	14.5 ± 0.1	13.8 ± 0.4	15.1	19.6	17.8 ± 0.3
140	Lauraceae	*Cinnamomum cassia* (L.) Presl	Grey bollywood	branch	15.2	14.6 ± 0.1	20 ± 0.2	18.1 ± 0.2	18.5 ± 0.6
148	Lycopodiaceae	*Diphasiastrum complanatum* (L.) Holub	Groundcedar	aerial part	20.8 ± 0.2	19.6 ± 0.1	21.7 ± 0.3	24.3 ± 0.3	22.6
151	Magnoliaceae	*Magnolia denudata* Desr.	Lilytree	bud of flower	15.2	16.1 ± 0.2	15.4 ± 0.2	17.2 ± 0.2	12.9
153	Malvaceae	*Bombax malabaricum* DC.	Bombax	root bark	10.3 ± 0.2	10.8 ± 0.2	10.4 ± 0.2	16.3	12.6
154		*Helicteres angustifolia* L.	Narrowleaf screwtree	root	17.7 ± 0.1	19.8	19.7 ± 0.5	26 ± 1	16.9 ± 0.1
155		*Pterospermum heterophyllum* Hance	Heterophyllous wingseedtree	root	15.7 ± 0.2	18.2 ± 0.1	19.3 ± 0.2	21.4 ± 0.4	18.2 ± 0.1
159	Meliaceae	*Melia azedarach* L.	Chinaberry tree	bark & root bark	18.4 ± 0.3	16.8 ± 0.3	22.2 ± 0.3	23.3 ± 0.6	19.8
168	Oleaceae	*Fraxinus fallax* Lingelsh.	Largeleaf Chinese ash	bark	13.4 ± 0.5	15.8 ± 0.3	20 ± 1	19.3 ± 0.7	19.5
169		*Jasminum nudiflorum* Lindl.	Winter jasmine	bud of flower	11.3	13.9	12.5 ± 0.1	15.9 ± 0.2	21.2 ± 0.8
172	Orchidaceae	*Nervilia fordii* Schltr.	Ford nervilla	rhizome & leaf	NIZ	8.3	NIZ	17.3	9.9 ± 0.1
173		*Pholidota chinensis* Lindl.	Chinese photinia herb	stem	14.9 ± 0.3	17 ± 0.4	17.3 ± 0.2	22.4 ± 0.8	17.2 ± 0.3
177	Orobanchaceae	*Striga asiatica* (L.) Kuntze	Asiatic witchweed	aerial part	11.9	11.1	8.8	19.4 ± 0.5	13.2
178	Paeoniaceae	*Paeonia lactiflora* Pall.	Chinese peony	root	15.6 ± 0.4	14.2 ± 0.1	17.4	16 ± 0.3	17.2 ± 0.2
179		*Paeonia suffruticosa* Andrews	Moutan peony	root bark	14.1 ± 0.1	18 ± 0.7	17 ± 0.3	18.2 ± 0.1	18.1 ± 0.2
180		*Paeonia veitchii* Lynch	Red Peony	root	14.5 ± 0.1	15.7 ± 0.1	15.8 ± 0.3	16.7	15.5 ± 0.7
182	Phyllanthaceae	*Phyllanthus emblica* L.	Emblic	fruit	16.6 ± 0.5	13.4 ± 0.3	19 ± 0.3	23.5 ± 0.2	18.6 ± 0.2
183	Pinaceae	*Pseudolarix amabilis* Rehder	Chinese golden larch	root bark	17.4 ± 0.5	17 ± 0.6	19.4 ± 0.4	17.4	19.7
185	Poaceae	*Bambusa tuldoides* Munro	Puntingpole bamboo	stem	9.4	9.3	8.1 ± 0.1	18.2 ± 0.3	9.7 ± 0.1
186		*Chrysopogon aciculatus* Trin.	Mackie’s pest	aerial part	11.4 ± 0.2	12.6 ± 0.4	12.3 ± 0.3	25 ± 1	14.2
192	Polygonaceae	*Polygonum bistorta* L.	Meadow bistort	rhizome	15.3 ± 0.2	15.7 ± 0.2	14.4 ± 0.4	19.2 ± 0.4	15.1 ± 0.1
193		*Polygonum chinense* L.	Chinese knotweed	aerial part	14.2 ± 0.1	13.6	14.2 ± 0.1	19 ± 0.5	17.2 ± 0.1
194		*Polygonum multiflorum* Thunb.	Tuber fleeceflower	stem	16.6 ± 0.3	15.2 ± 0.4	16.6 ± 0.3	21 ± 1	16.4 ± 0.1
195		*Polygonum multiflorum* Thunb.	Tuber fleeceflower	tuberous root	14 ± 1	17.7 ± 0.4	14.7 ± 0.5	19.5 ± 0.3	14.9
196		*Rumex obtusifolius L.*	Bitter dock	root	12.8	13 ± 0.2	18	22.9 ± 0.3	13.9
197	Primulaceae	*Ardisia japonica* Blume	Marlberry	aerial part	16.5 ± 0.6	12.4	13.2	19.2 ± 0.1	19 ± 1
198		*Lysimachia christinae* Hance	Herba lysimachiae	aerial part	14.3 ± 0.3	14.3 ± 0.2	16.4	27 ± 2	16.2 ± 0.1
200	Ranunculaceae	*Coptis chinensis* Franch.	Chinese goldthread	rhizome	23 ± 1	23.2 ± 0.4	22.5 ± 0.5	23 ± 1	22.3 ± 0.1
201		*Thalictrum aquilegifolium* L.	French meadow-rue	rhizome & root	14.4	15.4 ± 0.2	17.5 ± 0.6	17 ± 0.3	15.4 ± 0.4
202	Rosaceae	*Agrimonia pilosa* Ledeb.	Herba agrimoniae	aerial part	16.5 ± 0.2	16 ± 0.4	16.9 ± 0.4	21.1	18.4 ± 0.4
203		*Duchesnea indica* (Andr.) Focke	Indian strawberry	aerial part	11.5	13.9 ± 0.4	13.8 ± 0.7	21.5 ± 0.2	15.4 ± 0.5
205		*Geum aleppicum* Jacq.	Aleppo avens	aerial part	15.1 ± 0.5	14.7 ± 0.1	16.7 ± 0.3	22.7 ± 0.3	17.3 ± 0.3
206		*Prunus mume* Siebold & Zucc.	Japanese apricot	fruit	12.1 ± 0.1	11.5	13.3 ± 0.1	16.3 ± 0.5	12.2 ± 0.9
210	Rubiaceae	*Serissa serissoides* (DC.) Druce	Snowrose	aerial part	16.1 ± 0.3	18.9	21.5 ± 0.5	25.9 ± 0.1	15.9 ± 0.4
217	Rutaceae	*Phellodendron chinense* C. K. Schneid.	Chinese corktree	bark	20 ± 1	19.5 ± 0.3	18.8 ± 0.5	22.3 ± 0.3	21.8
218		*Zanthoxylum nitidum* DC.	Shiny-leaf prickly-ash	root	15.5 ± 0.4	17.7 ± 0.1	15.6	21 ± 1	14.5 ± 0.2
223	Saxifragaceae	*Saxifraga stolonifera* Meerb.	Creeping saxifrage	aerial part	11.5	11.6	11.6 ± 0.4	14.6 ± 0.2	13.3 ± 0.2
227	Solanaceae	*Lycium chinense* Mill.	Chinese boxthorn	root bark	19 ± 0.2	19.6 ± 0.1	22.8 ± 0.1	24 ± 1	21.4
230	Tamaricaceae	*Tamarix chinensis* Lour.	China tamarisk	branch & leaf	13.6 ± 0.2	14.4 ± 0.1	13.7 ± 0.4	16.6 ± 0.2	19.2
231	Thymelaeceae	*Daphne genkwa* Siebold & Zucc.	Chinese daphne	bud of flower	22.1 ± 0.1	23 ± 1	11.4 ± 0.3	23.7 ± 0.1	25.8 ± 0.4
239	Zingberaceae	*Curcuma phaeocaulis* Valeton	Rhizoma zedoariae	rhizome	14.1 ± 0.4	17.8 ± 0.3	16.5	22.7 ± 0.4	16.8 ± 0.3
	Ampicillin				21.7 ± 0.3	18.7 ± 0.5	18.5	20.2 ± 0.7	21.1 ± 0.5
	Oxacillin				15.5 ± 0.6	11.9 ± 0.7	13.3 ± 0.5	13.2 ± 0.4	18.2 ± 0.4
	DMSO				NIZ	NIZ	NIZ	NIZ	NIZ

^1^ 74 TCMP extracts (100 mg/mL) with strong inhibitory effects on the growth of reference *S. aureus* strain ATCC 25923 and antibiotic-resistant *S. aureus* strain SJTUF 20827 (DIZ ≥ 15 mm, Table S1 in Supplementary Material), were selected to investigate the wide range of antibacterial activities against multidrug-resistant *S. aureus* strains SJTUF 20745, 20746, 20758, 20978, and 20991. Diameter of inhibition zone (DIZ) was determined by the agar diffusion method in triplicate and value of DIZ was expressed as mean ± standard deviation (SD). DIZ values less than 8.0 mm was defined as “no inhibition zone (NIZ)”. Ampicillin (32 µg/mL) and oxacillin (4 µg/mL) were used as positive controls, while DMSO was used as a negative control.

**Table 2:** Minimal inhibitory concentration (MIC, mg/mL) and minimal bactericidal concentration (MBC, mg/mL) of selected TCMP extracts against multidrug-resistant *S. aureus*.

No.	Scientific Name	*S. aureus*													
		ATCC 25923		SJTUF 20745		SJTUF 20746		SJTUF 20758		SJTUF 20827		SJTUF 20978		SJTUF 20991	
		MIC	MBC	MIC	MBC	MIC	MBC	MIC	MBC	MIC	MBC	MIC	MBC	MIC	MBC
		(mg/mL)		(mg/mL)		(mg/mL)		(mg/mL)		(mg/mL)		(mg/mL)		(mg/mL)	
3	*Rhus chinensis* Mill.	0.1	3.125	0.1	3.125	0.195	3.125	0.1	0.78	0.1	0.39	0.195	3.125	0.195	1.56
22	*Ilex rotunda* Thunb.	0.78	3.125	1.56	25	1.56	> 25	1.56	6.25	1.56	12.5	0.78	12.5	0.78	12.5
51	*Inula japonica* Thunb.	3.125	6.25	1.56	> 25	3.125	25	3.125	> 25	3.125	6.25	1.56	6.25	3.125	6.25
58	*Leontice kiangnanensis* P.L.Chiu	0.78	1.56	0.78	25	0.78	> 25	0.78	3.125	6.25	25	0.78	12.5	0.78	3.125
61	*Oroxylum indicum* Vent.	0.39	1.56	0.39	6.25	0.78	25	0.78	1.56	0.39	0.78	1.56	3.125	1.56	3.125
65	*Isatis tinctoria* L.	3.125	25	3.125	> 25	6.25	> 25	6.25	12.5	12.5	12.5	6.25	> 25	12.5	> 25
81	*Terminalia chebula* Retz.	0.195	> 25	0.78	> 25	0.39	> 25	0.78	25	0.78	25	0.78	25	0.39	25
96	*Speranskia tuberculata* Baill.	0.78	25	0.78	> 25	0.78	12.5	0.78	12.5	0.78	12.5	1.56	12.5	0.78	12.5
97	*Acacia catechu* (L.f.) Willd.	0.195	3.125	0.39	6.25	0.195	6.25	0.78	0.78	0.78	1.56	0.195	0.78	0.195	1.56
103	*Dalbergia odorifera* T.C.Chen	0.39	3.125	0.39	12.5	0.39	6.25	0.39	3.125	0.39	6.25	0.39	0.78	0.39	6.25
112	*Quercus infectoria* Oliv.	0.1	12.5	0.195	25	0.195	6.25	0.195	12.5	0.19	1.56	0.195	1.56	0.195	12.5
115	*Spatholobus suberectus* Dunn	0.195	3.125	1.56	> 25	0.78	6.25	0.39	6.25	0.78	3.125	0.78	0.78	0.78	12.5
132	*Rabdosia rubescens* (Hemsl.) H.Hara	1.56	12.5	1.56	> 25	1.56	12.5	1.56	6.25	1.56	3.125	0.78	1.56	1.56	12.5
133	*Salvia miltiorrhiza* Bunge	0.39	3.125	1.56	25	3.125	12.5	1.56	6.25	0.78	3.125	3.125	6.25	3.125	6.25
168	*Fraxinus fallax* Lingelsh.	1.56	> 25	1.56	> 25	1.56	25	3.125	12.5	1.56	12.5	3.125	12.5	3.125	25
200	*Coptis chinensis* Franch.	0.39	1.56	0.195	25	0.39	12.5	0.39	1.25	0.39	0.78	0.195	1.56	0.195	0.78
202	*Agrimonia pilosa* Ledeb.	0.1	1.56	0.1	6.25	0.195	1.56	0.78	3.125	0.39	1.56	0.195	6.25	0.1	1.56
217	*Phellodendron chinense* C.K. Schneid.	0.78	3.125	0.78	12.5	0.39	1.56	0.78	1.56	0.78	0.78	0.195	3.125	0.39	1.56
	Ampicillin (μg/mL)	0.05	>25	6.25	> 25	>25	NA ^1^	>25	NA	>25	NA	3.125	>25	0.1	>25
	Oxacillin (μg/mL)	0.195	> 25	0.39	> 25	0.39	> 25	0.39	25	0.195	12.5	0.195	> 25	0.195	25

18 selected TCMP extracts with the highest DIZ values (DIZ ≥ 20 mm) were subjected to investigations of their MIC and MBC against one reference (ATCC 25923), one erythromycin-resistant (SJTUF 20827) and five multidrug-resistant (SJTUF 20745, SJTUF 20746, SJTUF 20758, SJTUF 20978 and SJTUF 20991) *S. aureus* strains (n = 3). Ampicillin and Oxacillin were used as positive controls. ^1^ NA, not applicable.

**Table 3:** Total phenolic content (TPC) and the total flavonoid content (TFC) in selected TCMP extracts.

No.	Scientific Name	TPC (mg GAE/g DW)	TFC (mg CE/g DW)
3	*Rhus chinensis* Mill.	632 ± 4 ^a^	36.8 ± 0.9 ^fg^
22	*Ilex rotunda* Thunb.	143 ± 3 ^fg^	16.9 ± 0.2 ^ijkl^
51	*Inula japonica* Thunb.	92 ± 6 ^i^	62 ± 3 ^e^
58	*Leontice kiangnanensis* P. L. Chiu	33 ± 1 ^k^	5.27 ± 0.08 ^l^
61	*Oroxylum indicum* Vent.	158 ± 3 ^f^	18.1 ± 0.6 ^ijk^
65	*Isatis tinctoria* L.	60 ± 3 ^j^	8.35 ± 0.2 ^kl^
81	*Terminalia chebula* Retz.	553 ± 4 ^b^	27 ± 0.8 ^ghi^
96	*Speranskia tuberculata* Baill.	31.4 ± 0.6 ^k^	13.4 ± 0.5 ^jkl^
97	*Acacia catechu* (L.f.) Willd.	545 ± 2 ^b^	377 ± 3 ^a^
103	*Dalbergia odorifera* T. C. Chen	215 ± 6 ^e^	62 ± 1 ^e^
112	*Quercus infectoria* Oliv.	646 ± 3 ^a^	38 ± 3 ^fg^
115	*Spatholobus suberectus* Dunn	489 ± 5 ^c^	214 ± 11 ^b^
132	*Rabdosia rubescens* (Hemsl.) H. Hara	135 ± 4 ^gh^	41 ± 1 ^f^
133	*Salvia miltiorrhiza* Bunge	56 ± 5 ^j^	34.5 ± 0.9 ^fgh^
168	*Fraxinus fallax* Lingelsh.	363 ± 15 ^d^	106 ± 5 ^d^
200	*Coptis chinensis* Franch.	95 ± 3 ^i^	23.1 ± 0.3 ^hij^
202	*Agrimonia pilosa* Ledeb.	371 ± 11 ^d^	154 ± 10 ^c^
217	*Phellodendron chinense* C. K. Schneid.	123 ± 2 ^h^	35 ± 2 ^fgh^

The experiments were performed in triplicate and the results were expressed as mean ± SD. One–way analysis of variance (ANOVA) plus *post hoc* Tukey test was performed and different superscript lowercase letters (a–l) indicated statistically significant difference (*p* < 0.05). *GAE*, *gallic acid equivalent*; *CE*, *catechin equivalent*; *DW*, *dry weight*.

**Table 4:** Cytotoxicity (LC_50_) on the human foreskin fibroblast (HFF) cells and selectivity index (SI) of selected TCMP extracts. SI value greater than 1 indicate that the extract is more toxic to the pathogen than to human cells, suggesting possible safety.

	Scientific Name	Cytotoxicity (LC_50_, µg/mL)	Selectivity Index (SI = LC_50_/MIC)
3	*Rhus chinensis* Mill.	77.6	0.77
22	*Ilex rotunda* Thunb.	>100	554
51	*Inula japonica* Thunb.	54.1	0.02
58	*Leontice kiangnanensis* P. L. Chiu	>100	2687
61	*Oroxylum indicum* Vent.	>100	NA
65	*Isatis tinctoria* L.	>100	76.0
81	*Terminalia chebula* Retz.	>100	2.20
96	*Speranskia tuberculata* Baill.	25.9	0.03
97	*Acacia catechu* (L.f.) Willd.	NA ^1^	NA
103	*Dalbergia odorifera* T. C. Chen	44.1	0.11
112	*Quercus infectoria* Oliv.	91.6	0.47
115	*Spatholobus suberectus* Dunn	>100	138
132	*Rabdosia rubescens* (Hemsl.) H. Hara	>100	158
133	*Salvia miltiorrhiza* Bunge	NA	NA
168	*Fraxinus fallax* Lingelsh.	>100	5.55
200	*Coptis chinensis* Franch.	>100	86.1
202	*Agrimonia pilosa* Ledeb.	>100	204
217	*Phellodendron chinense* C. K. Schneid.	>100	1.85

^1^ NA, not applicable.

**Table 5:** List of antibiotic-resistant *S. aureus* strains used in this study and their antibiotic resistance profiles [54].

*S. aureus* Strain Name	Antibiotic Resistance Profile
SJTUF 20745	Streptomycin, ciprofloxacin, clindamycin, erythromycin
SJTUF 20746	Gentamicin, ciprofloxacin, clindamycin, erythromycin
SJTUF 20758	Penicillin, streptomycin, clindamycin, erythromycin
SJTUF 20827	Erythromycin
SJTUF 20978	Ciprofloxacin, erythromycin, sulfisoxazole
SJTUF 20991	Ciprofloxacin, clindamycin, erythromycin, tetracycline

## Supplementary Materials

The following are available online at https://www.mdpi.com/2076-0817/9/3/185/s1, Table S1: Screening 239 TCMP extracts for antibacterial activities against reference *S. aureus* ATCC 25923 and antibiotic-resistant *S. aureus* SJTUF 20827 based on diameter inhibition zone (DIZ, mm), Figure S1: Dose-response curves for the viability of HFF cells exposed to selected TCMP extracts.
