# Supplementary material for: Large-Scale Screening of 239 Traditional Chinese Medicinal Plant Extracts for Their Antibacterial Activities against Multidrug-Resistant Staphylococcus aureus and Cytotoxic Activities
Source: Pathogens. 2020 Mar 4;9(3):185. doi: 10.3390/pathogens9030185 (PMC7157549; doi:10.3390/pathogens9030185)
Supplement: Supplementary file 1 [file pathogens-09-00185-s001.pdf]

pathogens

MDPI

Table S1. Screening 239 TCMP extracts for antibacterial activities against reference *S. aureus* ATCC 25923 and antibiotic-resistant *S. aureus* SJTUF 20827 based on diameter inhibition zone (DIZ, mm).

	Family	Scientific Name	Common Name	Extracted Plant Part	DIZ (mm) *S. aureus* ATCC 25923	DIZ (mm) *S. aureus* SJTUF 20827
1	Acanthaceae	*Andrographis paniculata* Nees	Green chireta	aerial part	8.4 ± 0.5	9.9 ± 0.3
2	Acoraceae	*Acorus tatarinowii* Schott	Grassleaf sweelflag	rhizome	9.5 ± 0.5	8.3 ± 0.2
3	Anacardiaceae	*Rhus chinensis* Mill.	Nutgall tree	gall	22 ± 0.5	20 ± 0.4
4		*Achyranthes aspera* L.	Chaffbur	aerial part	NIZ	NIZ
5	Amaranthaceae	*Gomphrena globosa* L.	Common globe amaranth	flower	8.1 ± 0.1	8.1 ± 0.3
6		*Kochia scoparia* (L.) Schrad.	Fireweed	fruit	11.2 ± 0.5	11.8 ± 0.4
7		*Bupleurum chinense* DC.	Chinese throughwax	root	NIZ	NIZ
8		*Cnidium monnieri* Cusson	Monnier's snowparsley	fruit	14 ± 0.1	NIZ
9		*Ferula sinkiangensis* K. M. Shen	Resina ferulae	resin	8.1 ± 0.2	NIZ
10		*Foeniculum vulgare* Mill.	Fennel	fruit	NIZ	NIZ
11		*Glehnia littoralis* F.Schmidt ex Miq.	American silvertop	root	8.2 ± 0.3	NIZ
12	Apiaceae	*Hydrocotyle sibthorpioides* Lam.	Lawn marshpennywort	aerial part	11.3 ± 0.1	8.5 ± 0.1
13		*Ligusticum sinense* Oliv.	Sichuan lovage	rhizome	10.6 ± 0.2	9.2 ± 0.4
14		*Notopterygium forbesii* H.Boiss.	Notopterygium	rhizome & root	10.9	9.3 ± 0.5
15		*Peucedanum praeruptorum* Dunn	Peucedanum	root	NIZ	8.4 ± 0.2
16		*Saposhnikovia divaricata* (Turcz.) Schischk.	Siler	root	NIZ	NIZ
17		*Cynanchum atratum* Bunge	Blackend Swallowwort	rhizome	NIZ	8.1 ± 0.5
18		*Cynanchum glaucescens* (Decne.) Handel-Mazzetti	Willowleaf swallowwort	rhizome & root	NIZ	8.5 ± 0.3
19	Apocynaceae	*Trachelospermum jasminoides* Lem.	Chinese star-jessamine	leaf & stem	12.5 ± 0.3	10.2 ± 0.5
20		*Cynanchum paniculatum* (Bunge) Kitagawa	Radix cynanchi paniculati	rhizome	11 ± 0.1	10 ± 0.6
21	Aquifoliaceae	*Ilex pubescens* Hook. & Arn.	Pubescent holly	root	13.3 ± 0.3	13.5 ± 0.3
22		*Ilex rotunda* Thunb.	Kurogane holly	bark	22.4 ± 0.2	20.7 ± 0.8
23		*Homalomena occulta* Schott	Rhizoma homalomenae	rhizome	NIZ	NIZ
24		*Pinellia ternata* (Thunb. ex Murray) T.Makino	Pinellia	tuberous root	NIZ	8.1 ± 0.1
25	Araceae	*Scindapsus aureus* (Linden & André) Engl.	Golden pothos	flower	8.9 ± 0.1	8.7 ± 0.1
26		*Spirodela polyrrhiza* (L.) Schleid.	Common duckweed	aerial part	10.9 ± 0.1	15
27		*Aralia chinensis* L.	Chinese angelica tree	bark	11.4 ± 0.7	9.7 ± 0.1
28	Araliaceae	*Panax ginseng* C.A.Mey.	Asian ginseng	root	NIZ	NIZ
29		*Panax notoginseng* (Burkill) F.H.Chen	South China ginseng	root	NIZ	NIZ
30	Arecaceae	*Daemonorops draco* (Willd.) Blume	Dragon's blood	resin	NIZ	NIZ

31	Aristolochiaceae	*Asarum heterotropoides* F.Schmidt	Manchurian wildginge	rhizome and root	12.7	14.7 ± 0.4
32	Asparagaceae	*Anemarrhena asphodeloides* Bunge	Rhizoma anemarrhena	tuberous root	NIZ	NIZ
33		*Asparagus cochinchinensis* Merr.	Chinese asparagus	tuberous root	8.2 ± 0.5	NIZ
34		*Ophiopogon japonicus* Ker Gawl.	Dwarf lilyturf	tuberous root	NIZ	8.2 ± 0.3
35		*Polygonatum cyrtonema* Hua	Rhizoma polygonati	rhizome	NIZ	NIZ
36	Asphodelaceae	*Aloe ferox* Mill.	Cape aloe	dried gel	15.2 ± 0.5	11.6 ± 0.3
37		*Hemerocallis citrina* Baroni	Citron daylily	aerial part	13.4 ± 0.1	11.1 ± 0.4
38	Asteraceae	*Arctium lappa* L.	Burdock	fruit	8.8 ± 0.3	8.2 ± 0.4
39		*Artemisia argyi* H.Lév. & Vaniot	Chinese mugwort	leaf	12.9 ± 0.2	12 ± 0.3
40		*Artemisia capillaris* Thunb.	Capillary artemisia	aerial part	9.8 ± 0.1	NIZ
41		*Artemisia scoparia* Waldst. & Kit.	Redstem wormwood	aerial part	11.6 ± 0.3	9.2 ± 0.7
42		*Bidens bipinnata* L.	Spanish needles	aerial part	NIZ	8.9 ± 0.2
43		*Centipeda minima* A.Braun & Asch.	Spreading sneezeweed	aerial part	11.5 ± 0.3	9.8 ± 0.2
44		*Chrysanthemum indicum* L.	Indian chrysanthemum	flower	13.8 ± 0.3	9.5 ± 0.5
45		*Eclipta prostrata* (L.) L.	Eclipta	aerial part	13.1 ± 0.2	13.3 ± 0.2
46		*Elephantopus scaber* L.	Cucha cara	aerial part	13.8 ± 0.6	14.6 ± 0.2
47		*Emilia sonchifolia* (L.) DC.	Lilac tasselflower	aerial part	8.9 ± 0.6	NIZ
48		*Eupatorium fortunei* Turcz.	Chinese eupatorium	aerial part	8.1 ± 0.3	8.2 ± 0.3
49		*Hemistepta lyrata* Bunge	Lyre-shape hemistepta	aerial part	14.1 ± 0.2	12.4
50		*Inula helenium* L.	Elecampane	root	11.8 ± 0.2	10.8 ± 0.4
51		*Inula japonica* Thunb.	Inula flower	flower (capitulum)	21 ± 1	22v± 1
52		*Kalimeris indica* (L.) Sch.Bip.	Indian aster	aerial part	11.3 ± 0.4	NIZ
53		*Rhaponticum uniflorum* DC.	Radix rhapontici	root	10 ± 0.4	11 ± 0.2
54		*Senecio scandens* (L.) Buch.-Ham.	Climbing groundsel herb	aerial part	NIZ	8.2 ± 0.3
55		*Xanthium sibiricum* Patrin	Herba xanthii	aerial part	8.1 ± 0.3	9.2 ± 0.4
56		*Codonopsis pilosula* Nannf.	Bellflower	root	8.2 ± 0.3	8.4 ± 0.1
57	Balsaminaceae	*Impatiens balsamina* L.	Balsam	seed	NIZ	12.3 ± 0.3
58	Berberidaceae	*Leontice kiangnanensis* P.L.Chiu	Rhizoma corydalis	tuberous root	20.5 ± 0.9	21.6 ± 0.3
59		*Mahonia fortunei* (Lindl.) Fedde	Fortune mahonia	leaf	11.3 ± 0.3	10.9 ± 0.3
60	Bignoniaceae	*Campsis grandiflora* K.Schum.	Chinese trumpet-creeper	flower	NIZ	NIZ
61		*Oroxylum indicum* Vent.	Indian trumpet flower	seed	21.5 ± 0.5	19.4 ± 0.5
62	Boraginaceae	*Lithospermum erythrorhizon* Siebold & Zucc.	Purple gromwell	leaf	13	14.5 ± 0.6
63	Brassicaceae	*Brassica juncea* (L.) Czern.	Chinese mustard	seed	NIZ	NIZ
64		*Isatis indigotica* Fortune	Woad	root	8.1 ± 0.2	8.6 ± 0.5
65		*Isatis tinctoria* L.	Dyer's woad	leaf	25 ± 2	22.4 ± 0.1
66		*Raphanus sativus* L.	Garden radish	seed	8.4 ± 0.4	NIZ
67	Burseraceae	*Commiphora myrrha* Engl.	Myrrh	resin	9 ± 0.2	9.5 ± 0.2

68	Buxaceae	*Buxus sinica* (Rehder & E.H.Wilson) M.Cheng	Chinese boxwood	leaf & branch	10.2 ± 0.5	9.6 ± 0.2
69	Cannabaceae	*Humulus scandens* (Lour.) Merr.	Japanese hop	leaf & stem	8.8 ± 0.3	NIZ
70	Campanulaceae	*Platycodon grandiflorus* A.DC.	Balloon flower	root	NIZ	8.2 ± 0.1
71	Caprifoliaceae	*Dipsacus japonicus* Miq.	Japanese teasel	root	NIZ	8.4 ± 0.2
72		*Lonicera japonica* Thunb.	Chinese honeysuckle	branch	9.3 ± 0.1	8.7 ± 0.2
73		*Lonicera maackii* (Rupr.) Maxim.	Amur honeysuckle	flower	8.6	NIZ
74		*Patrinia scabiosifolia* Fisch.	Herba patriniae	aerial part	11.5 ± 0.5	NIZ
75	Caryophyllales	*Vaccaria hispanica* (Miller) Rauschert	Cowcockle	seed	8.5 ± 0.3	NIZ
76	Celastraceae	*Euonymus fortunei* (Turcz.) Hand.-Mazz.	Climbing euonymus	leaf and stem	10.9 ± 0.4	11.4 ± 0.1
77	Chloranthaceae	*Sarcandra glabra* (Thunb.) Nakai	Herba sarcandrae	branch	11.9 ± 0.1	8.2 ± 0.3
78	Cibotiaceae	*Cibotium barometz* (L.) J.Sm.	Scythian-lamb	rhizome	11.3 ± 0.2	NIZ
79	Convolvulaceae	*Dichondra repens* J.R.Forst. & G.Forst.	Dichondra	aerial part	13.5 ± 0.3	12 ± 2
80	Combretaceae	*Quisqualis indica* L.	Rangoon creeper	fruit	NIZ	NIZ
81		*Terminalia chebula* Retz.	Myrobalan	Fruit	21.6 ± 0.7	22 ± 1
82	Crassulaceae	*Sedum sarmentosum* Bunge	Stringy stonecrop	aerial part	NIZ	NIZ
83	Cucurbitaceae	*Momordica grosvenorii* Swingle	Buddhafruit	fruit	NIZ	8.8 ± 0.2
84		*Trichosanthes kirilowii* Maxim.	Chinese snake gourd	root	9.1 ± 0.2	NIZ
85				fruit	NIZ	NIZ
86				fruit peel	8.3 ± 0.2	8.4 ± 0.1
87	Dioscoreaceae	*Dioscorea bulbifera* L.	aerial yam	tuberous root	22 ± 1	18.5 ± 0.2
88		*Dioscorea hypoglauca* Palib.	Mountain yam	rhizome	8.2 ± 0.1	8.2 ± 0.1
89	Dryopteridaceae	*Dryopteris crassirhizoma* Nakai	Japanese flowering fern	rhizome	8.1 ± 0.3	NIZ
90	Ebenaceae	*Diospyros kaki* Thunb.	Chinese persimmon	calyx	18 ± 1	13 ± 2
91	Elaeagnaceae	*Elaeagnus pungens* Thunb.	Spiny oleaster	leaf	10.1 ± 0.4	12.3 ± 0.2
92	Ericaceae	*Pyrola calliantha* Andres	Chinese pyrola	aerial part	14.5 ± 0.2	14.7 ± 0.4
93	Euphorbiaceae	*Euphorbia helioscopia* L.	Summer spurge	aerial part	13.7 ± 0.2	10.8 ± 0.6
94		*Euphorbia hirta* L.	Garden spurge	aerial part	13.4 ± 0.2	10 ± 0.3
95		*Euphorbia humifusa* Willd.	Herba euphorbiae humifusae	aerial part	14.1 ± 0.4	13.2 ± 0.2
96		*Speranskia tuberculata* Baill.	Herba speranskiae tuberculatae	aerial part	27 ± 1	25 ± 1
97	Fabaceae	*Acacia catechu* (L.f.) Willd.	Catechu	branch	24.5 ± 0.5	19.8 ± 0.9
98		*Albizia julibrissin* Durazz.	Silk tree	flower	8.6 ± 0.4	NIZ
99				bark	8.7 ± 0.4	11.1 ± 0.7
100		*Caesalpinia minax* Hance	Semen caesalpiniae	seed	12.1 ± 0.2	9.7 ± 0.4
101		*Cassia occidentalis* L.	Coffee senna	seed	15.3 ± 0.4	14.2 ± 0.2
102		*Cassia tora* L.	Sickle Senna	seed	13.2 ± 0.5	14.6 ± 0.3
103		*Dalbergia odorifera* T.C.Chen	Fragrant rosewood	heart wood & trunk	19.3 ± 0.4	21.1 ± 0.2
104		*Erythrina variegata* var. orientalis (L.) Merr.	Tiger's claw	bark	11.5 ± 0.4	11.6 ± 0.1

105		*Flemingia prostrata* Roxb.f. ex Roxb.	Philippine flemingia	root	12.4 ± 0.2	13.5 ± 0.2
106		*Gleditsia sinensis* Lam.	Chinese honey locust	fruit	13.2 ± 0.1	9.9 ± 0.4
107				branch	16.4 ± 0.3	13.9 ± 0.2
108		*Glycine max* (L.) Merr.	Soya bean	seed	11.4 ± 0.2	NIZ
109		*Glycyrrhiza uralensis* Fisch.	Licorice	rhizome & root	14.3 ± 0.3	14.1 ± 0.1
110		*Lablab purpureus* (L.) Sweet	Lablab Bean	seed	20.4 ± 0.2	15 ± 0.2
111		*Mimosa pudica* L.	Sensitive grass	aerial part	13.1 ± 0.2	11.6 ± 0.4
112		*Quercus infectoria* Oliv.	Aleppo oak	gall	24 ± 2	21 ± 1
113		*Sophora alopecuroides* L.	Foxtail-like sophora	seed	8.3 ± 0.2	8.3 ± 0.5
114		*Sophora tonkinensis* Gagnepain	Vietnamese sophora	rhizome & root	14.8 ± 0.3	12.8
115		*Spatholobus suberectus* Dunn	Caulis spatholobi	stem	21.2	21.6 ± 0.6
116	Ganodermataceae	*Ganoderma lucidum* (Curtis) P.Karst.	Reishi mushroom	whole mushroom	9.5	11.3 ± 0.5
117	Gelsemiaceae	*Gelsemium elegans* Benth.	Graceful jessamine	root	8.3 ± 0.1	NIZ
118	Gentianaceae	*Gentiana macrophylla* Pall.	Large-leaf gentian	root	8.8 ± 0.6	8.2 ± 0.21
119		*Gentiana scabra* Bunge	Scabrous gentian	rhizome & root	NIZ	NIZ
120		*Tripterospermum affine* (Wall. ex C.B.Cl.) H.Smith	Herba tripterospermum	aerial part	8.4 ± 0.2	8.3 ± 0.2
121	Ginkgoaceae	*Ginkgo biloba* L.	Ginkgo	seed	8.1 ± 0.5	NIZ
122	Hypericaceae	*Hypericum japonicum* Thunb.	Matted St. John's-wort	aerial part	21.1 ± 0.3	18.8 ± 0.3
123	Hypoxidaceae	*Curculigo orchioides* Gaertn.	Golden eye-grass	rhizome	13.3 ± 0.1	10.9 ± 0.5
124	Lamiaceae	*Clerodendrum bungei* Steud.	Glory-flower	aerial part	10.1 ± 0.2	9.9 ± 0.1
125		*Glechoma longituba* (Nakai) Kuprian.	Longtube ground ivy herb	aerial part	10.8 ± 0	11 ± 0.1
126		*Isodon serra* Kudo	Herba rabdosiae	aerial part	19.1 ± 0.3	16.5 ± 0.3
127		*Leonurus japonicus* Houtt.	Chinese motherwort	aerial part	12.7 ± 0.4	10.3 ± 0.2
128		*Lycopus lucidus* var. hirtus (Regel) Makino & Nemoto	Herba lycopi	aerial part	10 ± 0.1	9.4 ± 0.2
129		*Mentha haplocalyx* Briq.	Chinese mint	aerial part	10.1 ± 0.2	8.3
130		*Mosla chinensis* Maxim.	Chinese mosla	aerial part	11.7 ± 0.2	11.6 ± 0.7
131		*Perilla frutescens* Britton.	Beefsteak mint	leaf	13.7 ± 0.5	10.5 ± 0.1
132		*Rabdosia rubescens* (Hemsl.) H.Hara	Blushred rabdosia	aerial part	22.2 ± 0.1	21 ± 1
133		*Salvia miltiorrhiza* Bunge	Chinese salvia	rhizome & root	25.7 ± 0.7	20.9 ± 0.2
134		*Schizonepeta tenuifolia* (Benth.) Briq.	Herba schizonepetae	aerial part	10 ± 0.3	8.8 ± 0.4
135		*Thymus serpyllum* L.	Brecklan thyme	seed	NIZ	8.13 ± 0.3
136		*Vitex negundo* L.	Chinese chastetree	fruit	8.5 ± 0.2	8.5 ± 0.5
137		*Vitex trifolia* L.	Indian wild pepper	fruit	12.6 ± 0.1	12.4 ± 0.3
138	Lardizabalaceae	*Akebia quinata* Decne.	Fiveleaf	stem	8.8 ± 0.1	NIZ
139		*Sargentodoxa cuneata* Rehder & E.H.Wilson	Sargentgloryvine	stem	17.9 ± 0.3	16.3
140	Lauraceae	*Cinnamomum cassia* (L.) Presl	Grey bollywood	branch	15 ± 0.5	11.2 ± 0.2
141		*Lindera aggregata* (Sims) Kosterm.	Combined spicebush	tuberous root	13.3 ± 0.4	9.8 ± 0.1

142		*Litsea cubeba* Pers.	Mountain-pepper	fruit	NIZ	NIZ
143	Liliaceae	*Fritillaria cirrhosa* D. Don	Sichuan fritillary	Bulb	NIZ	NIZ
144		*Fritillaria thunbergii* Miq.	Fritillary	Bulb	NIZ	9.1 ± 0.5
145		*Fritillaria usuriensis* Maxim.	Unibract fritillary	bulb & rhizome	NIZ	NIZ
146	Lindsaeaceae	*Stenoloma chusanum* (L.) Ching	Common wedgelet fern	aerial part	13.9 ± 0.3	12.6 ± 0.2
147	Lycoperdaceae	*Calvatia lilacina* (Mont. & Berk.) Lloyd	Lasiosphaera seu calvatia	whole mushroom	8.2 ± 0.4	8.2 ± 0.2
148		*Diphasiastrum complanatum* (L.) Holub	Groundcedar	aerial part	22.7 ± 0.4	17.6 ± 0.1
149	Lygodiaceae	*Lygodium japonicum* (Thunb.) Sw.	Japanese climbing fern	spore	8.3 ± 0.3	NIZ
150	Lythraceae	*Punica granatum* L.	Pomegranate	fruit peel	13.1 ± 0.4	13.7 ± 0.5
151	Magnoliaceae	*Magnolia denudata* Desr.	Lilytree	bud of flower	10.5 ± 0.2	14.8 ± 0.1
152	Malvaceae	*Abutilon indicum* (L.) Sweet	Indian abutilon	aerial part	NIZ	8.4 ± 0.3
153		*Bombax malabaricum* DC.	Bombax	root bark	16.2 ± 0.3	11.5 ± 0.2
154		*Helicteres angustifolia* L.	Narrowleaf screwtree	root	18.4	17.5 ± 0.1
155		*Pterospermum heterophyllum* Hance	Heterophyllous wingseedtree	root	18.7	17 ± 0.1
156		*Sterculia lychnophora* Hance	Boat-fruited sterculia	reed	11.8 ± 0.1	10.2 ± 0.4
157		*Urena lobata* L.	Caesar weed	aerial part	9.3 ± 0.5	8.5 ± 0.2
158	Melanthiaceae	*Paris polyphylla* Sm.	Rhizoma paridis	rhizome	NIZ	NIZ
159	Meliaceae	*Melia azedarach* L.	Chinaberry tree	bark & root bark	21.1 ± 0.3	18.1 ± 0.3
160		*Melia toosendan* Sieb.&Zucc.	Cape-lilac	fruit	NIZ	8.3 ± 0.2
161	Menispermaceae	*Stephania delavayi* Diels	Epigeal Srephaia	tuberous root	10.5 ± 0.1	9 ± 0.5
162		*Stephania tetrandra* S.Moore	Stephania root	root	10.6 ± 0.6	12.9 ± 0.4
163		*Tinospora capillipes* Gagnep.	Arrowshaped tinospora	tuberous root	9.0 ± 0.2	8.3 ± 0.2
164	Moraceae	*Broussonetia papyrifera* Vent.	Paper mulberry	fruit	8.2 ± 0.1	NIZ
165		*Morus alba* L.	White mulberry	fruit	NIZ	NIZ
166	Myristicaceae	*Myristica fragrans* Houtt.	Mace	seed	9.2 ± 0.2	9.9 ± 0.1
167	Oleaceae	*Forsythia suspensa Vahl*	Forsythia	fruit	12.9 ± 0.3	9.4 ± 0.6
168		*Fraxinus fallax* Lingelsh.	Largeleaf chinese ash	bark	16.8 ± 0.3	21 ± 1
169		*Jasminum nudiflorum* Lindl.	Winter jasmine	bud of flower	14.4 ± 0.2	12.8 ± 0.3
170	Orchidaceae	*Bletilla striata* Rchb.f.	Chinese ground orchid	tuberous root	8.3 ± 0.1	8.9 ± 0.1
171		*Cremastra appendiculata* (D.Don) Makino	Pseudobulbus cremastrae	bulb	12 ± 0.3	11.3 ± 0.2
172		*Nervilia fordii* Schltr.	Ford nervilla	rhizome and leaf	14.7 ± 0.5	9.25
173		*Pholidota chinensis* Lindl.	Chinese Photinia herb	stem	17.5 ± 0.2	18.5 ± 0.1
174	Orobanchaceae	*Cistanche deserticola* Y.C.Ma	Desert-broomrape	succulent stem	9.2 ± 0.4	9.2 ± 0.2
175		*Phtheirospermum japonicum* (Thunb.) Kanitz	Capillary wormwood herb	aerial part	NIZ	10.8 ± 0.5
176		*Rehmannia glutinosa* (Gaertn.) Libosch.	Radix rehmanniae preparta	tuberous root	NIZ	NIZ
177		*Striga asiatica* (L.) Kuntze	Asiatic witchweed	aerial part	14.6 ± 0.3	12.9 ± 0.2
178	Paeoniaceae	*Paeonia lactiflora* Pall.	Chinese peony	root	18.6 ± 0.1	15.9 ± 0.5

179		*Paeonia suffruticosa* Andrews	Moutan peony	root bark	18 ± 0.2	17 ± 1
180		*Paeonia veitchii* Lynch	Red Peony	root	19.1 ± 0.2	17.3 ± 0.3
181	Papaveraceae	*Corydalis decumbens* Pers.	Rhizoma corydalis decumbentis	rhizome	NIZ	8.4 ± 0.3
182	Phyllanthaceae	*Phyllanthus emblica* L.	Emblic	fruit	20 ± 0.1	17 ± 1
183	Pinaceae	*Pseudolarix amabilis* Rehder	Chinese golden larch	root bark	17.5 ± 0.4	15.3 ± 0.1
184	Piperaceae	*Piper wallichii* Hand.-Mazz.	Angiosperms	leaf and stem	8.28 ± 0.1	9.1 ± 0.3
185	Poaceae	*Bambusa tuldoides* Munro	Puntingpole bamboo	stem	14.2 ± 0.3	12.6
186		*Chrysopogon aciculatus* Trin.	Mackie's pest	aerial part	15.3 ± 0.1	9.4
187		*Coix lacryma-jobi* L.	Adlay	seed	8.1 ± 0.1	8.6 ± 0.3
188		*Cymbopogon distans* (Nees ex Steud.) Will.Watson	Remote Lemongrass	aerial part	13.2	11.3 ± 0.3
189		*Phragmites communis* Trin.	European reed	rhizome	10.4 ± 0.3	8.4 ± 0.2
190	Polygalaceae	*Polygala japonica* Houtt.	Japanese milkwort	aerial part	NIZ	8.6 ± 0.6
191		*Poria cocos* F.A.Wolf	Indian buead	sclerotium	NIZ	NIZ
192	Polygonaceae	*Polygonum bistorta* L.	Meadow bistort	rhizome	17.2 ± 0.2	16 ± 1
193		*Polygonum chinense* L.	Chinese knotweed	aerial part	17.2 ± 0.5	13
194		*Polygonum multiflorum* Thunb.	Tuber fleeceflower	stem	18.2 ± 0.5	15.5 ± 0.3
195				tuberous root	16.2 ± 0.4	12.6 ± 0.1
196		*Rumex obtusifolius L.*	Bitter dock	root	14.7 ± 0.7	13 ± 0.5
197	Primulaceae	*Ardisia japonica* Blume	Marlberry	aerial part	14.3	13.4
198		*Lysimachia christinae* Hance	Herba lysimachiae	aerial part	17.6 ± 0.4	12.1 ± 0.3
199	Ranunculaceae	*Cimicifuga foetida* L.	Chinese cimicifuga	rhizome	9 ± 0.3	9.9 ± 0.2
200		*Coptis chinensis* Franch.	Chinese goldthread	rhizome	21.7 ± 0.5	27 ± 1
201		*Thalictrum aquilegifolium* L.	French meadow-rue	rhizome & root	12.7	17.8 ± 0.2
202	Rosaceae	*Agrimonia pilosa* Ledeb.	Herba agrimoniae	aerial part	20 ± 1	19 ± 1
203		*Duchesnea indica* (Andr.) Focke	Indian strawberry	aerial part	15.2 ± 0.4	11 ± 0.3
204		*Eriobotrya japonica* (Thunb.) Lindl.	Japanese medlar	leaf	12.1 ± 0.4	10.6 ± 0.1
205		*Geum aleppicum* Jacq.	Aleppo avens	aerial part	18 ± 0.4	13.5 ± 0.2
206		*Prunus mume* Siebold & Zucc .	Japanese apricot	fruit	14.6 ± 0.1	13.8 ± 0.1
207		*Rosa laevigata* Michx.	Cherokee rose	fruit	11.4 ± 0.5	10.4 ± 0.4
208	Rubiaceae	*Gardenia jasminoides* Ellis	Cape jasmine	fruit	9.1 ± 0.4	NIZ
209		*Morinda officinalis* F.C.How	Indian mulberry	root	8.1 ± 0.2	8.5 ± 0.1
210		*Serissa serissoides* (DC.) Druce	Snowrose	aerial part	14.5 ± 0.4	15.7 ± 0.1
211		*Uncaria sinensis* Havil.	Gambir plant	branch	13.2 ± 0.5	11.7 ± 0.2
212	Rutaceae	*Citrus medica* var. sarcodactylis (Noot.) Swingle	flesh-finger citron	fruit	8.3 ± 0.1	8.4 ± 0.5
213		*Citrus reticulata* Blanco	Mandarin	fruit peel	NIZ	NIZ
214		*Dictamnus dasycarpus* Turcz.	Dense-fruit dittany	root bark	8.6 ± 0.1	8.2 ± 0.2

215		*Evodia lepta* (Spreng.) Merr.	Thin evodia twig and leaf	root	10.6 ± 0.3	10 ± 1
216		*Evodia ruticarpa* (A.Juss.) Hook.f. & Thomson	Evodia	fruit	11.7 ± 0.2	10.4 ± 0.4
217		*Phellodendron chinense C.K.*Schneid.	Chinese corktree	bark	18.1 ± 0.2	23.2 ± 0.6
218		*Zanthoxylum nitidum* DC.	Shiny-leaf prickly-ash	root	13.8	15.8
219	Santalaceae	*Thesium chinense* Turcz.	Chinese bastardtoadflax herb	aerial part	8.2 ± 0.2	10 ± 0.1
220		*Viscum coloratum* Nakai	Herba visci	leaf & branch	8.7 ± 0.2	8.1 ± 0.2
221	Sapindaceae	*Dimocarpus longan* Lour.	Longan	seed coat	8.1	8.3 ± 0.1
222	Saururaceae	*Saururus chinensis* (Lour.) Baill.	Chinese lizardtail	aerial part	8.4 ± 0.4	9.9 ± 0.6
223	Saxifragaceae	*Saxifraga stolonifera* Meerb.	Creeping saxifrage	aerial part	15 ± 0.2	10.6 ± 0.2
224	Simaroubaceae	*Brucea javanica* (L.) Merr.	Java brucea	fruit	8.5 ± 0.3	8.7 ± 0.2
225	Solanaceae	*Datura stramonium* L.	Jamestown weed	flower	10.3 ± 0.8	10.7 ± 0.6
226		*Lycium barbarum* L.	Chinese boxthorn	fruit	NIZ	NIZ
227		*Lycium chinense* Mill.	Chinese boxthorn	Root bark	16.8 ± 0.3	13.6
228		*Solanum lyratum* Thunb.	Herba solani lyrati	aerial part	9 ± 0.3	8.6 ± 0.3
229	Stemonaceae	*Stemona sessilifolia* (Miq.) Miq.	Stemona	root	NIZ	8.3 ± 0.1
230	Tamaricaceae	*Tamarix chinensis* Lour.	China tamarisk	branch & leaf	16.3 ± 0.1	16.5 ± 0.3
231	Thymelaeaceae	*Daphne genkwa* Siebold & Zucc.	Chinese daphne	bud of flower	20.3 ± 0.5	16.8 ± 0.2
232		*Wikstroemia indica* C.A.Mey.	Tiebush	root	13 ± 0.4	13 ± 1
233	Typhaceae	*Sparganium stoloniferum* Buch.-Ham.	Common burreed	tuberous root	9.3 ± 0.2	NIZ
234		*Typha angustifolia* L.	Cattail	pollen	8.5 ± 0.4	NIZ
235	Verbenaceae	*Verbena officinalis* L.	Common verbena	aerial part	11.9 ± 0.1	11.9 ± 0.3
236	Vitaceae	*Ampelopsis japonica* Makino	Japanese peppervine	tuberous root	12.1 ± 0.4	10.6 ± 0.1
237		*Parthenocissus tricuspidata* Planch.	Japanese-creeper	root & stem	8.3	8.2 ± 0.1
238	Zingiberaceae	*Curcuma longa* L.	Common turmeric	rhizome	8.2 ± 0.2	8.6 ± 0.1
239		*Curcuma phaeocaulis* Valeton	Rhizoma zedoariae	rhizome	18.5 ± 0.6	12.9 ± 0.4
	Ampicillin				34.6 ± 0.3	20.8 ± 0.7
	Oxacillin				17.0 ± 0.5	12.9 ± 0.4
	DMSO				NIZ	NIZ

Inhibitory effects of 239 TCMP extracts were determined by DIZ in triplicate. Value of DIZ was expressed as mean ± standard deviation (SD). DIZ values less than 8.0 mm was defined as no inhibition zone (NIZ). Ampicillin (32 µg/mL) and oxacillin (4 µg/mL) were used as positive controls, while DMSO was used as a negative control.

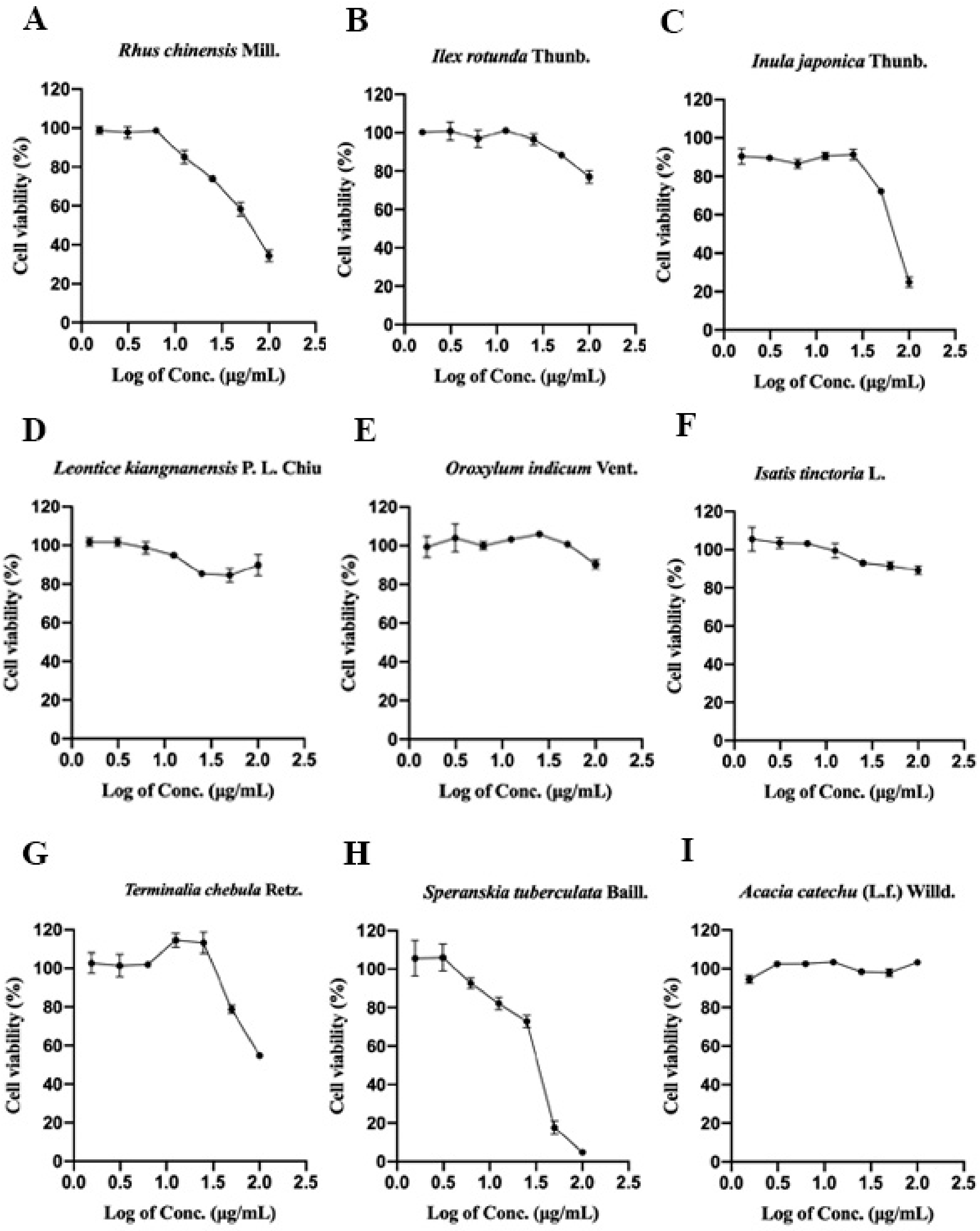
A
Rhus chinensis Mill.
B
Ilex rotunda Thunb.
C
Inula japonica Thunb.
D
Leontice kiangnanensis P. L. Chiu
E
Oroxylum indicum Vent.
F
Isatis tinctoria L.
G
Terminalia chebula Retz.
H
Speranskia tuberculata Baill.
I
Acacia catechu (L.f.) Willd.
Cell viability (%)
Log of Conc. (μg/mL)
0
20
40
60
80
100
120
0.0
0.5
1.0
1.5
2.0
2.5

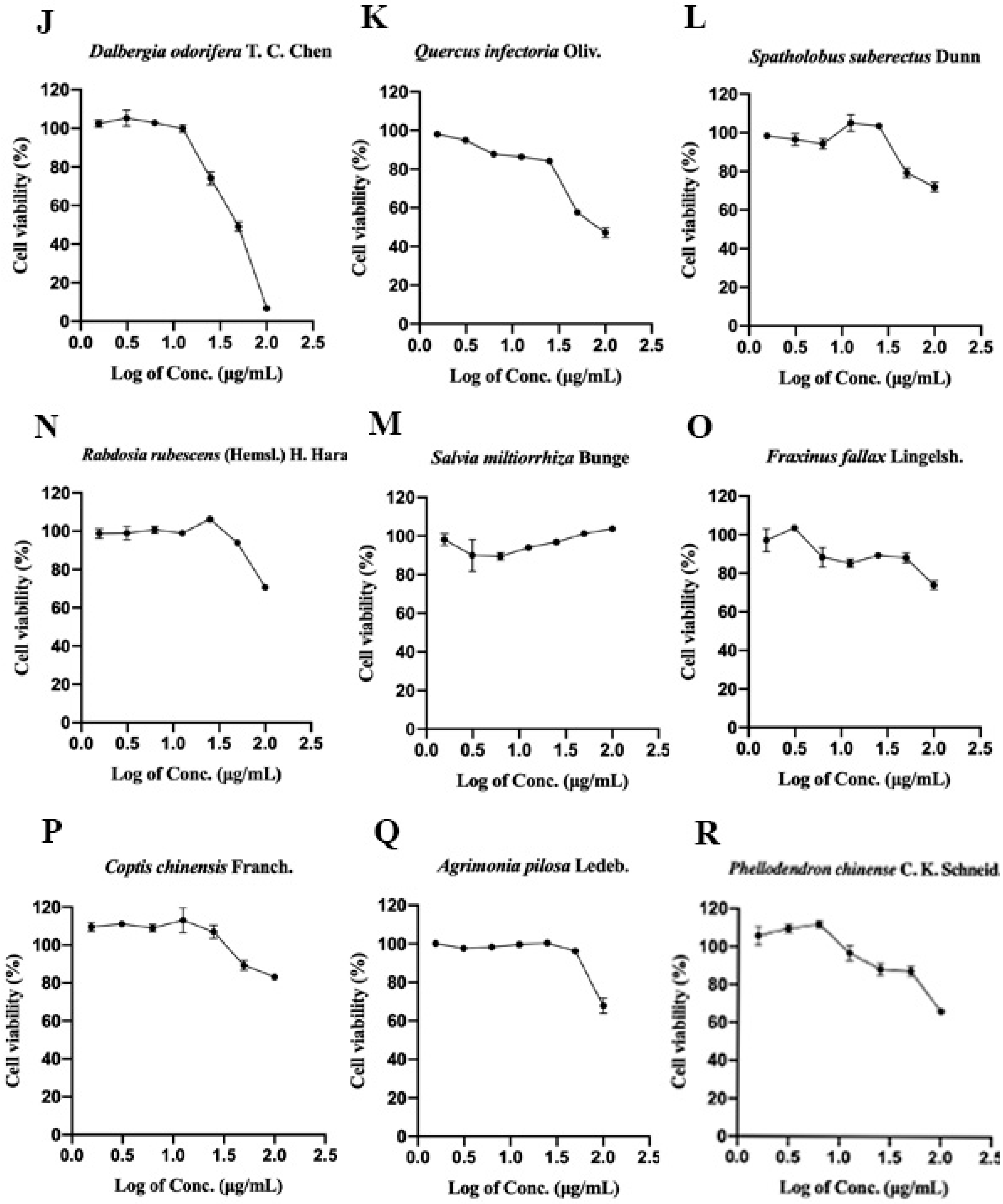

Figure S1. Dose-response curves for the cell viability in HFF cells exposed to TCMP extracts: (A) *R. chinensis*; (B) *I. rotunda*; (C) *I. japonica*; (D) *L. kiangnanensis*; (E) *O. indicum*; (F) *I. tinctorial*; (G) *T. chebula*; (H) *S. tuberculate*; (I) *A. catechu*; (J) *D. odorifera*; (K) *Q. infectoria*; (L) *S. suberectus*; (N) *R. rubescens*; (M) *S. miltiorrhiza*; (O) *F. fallax*; (P) *C. chinensis*; (Q) *A. Pilosa*; (R) *P. chinense.* The graphs represent the percentage of cell viability versus logarithm of the concentration.
